# Microtubule-Associated Proteins: From Dynamic Regulation of Microtubules to Cellular Architecture

**DOI:** 10.3390/cells15141289

**Published:** 2026-07-18

**Authors:** Eva Pais, Kenneth Bødtker Schou

**Affiliations:** Danish Cancer Institute, Strandboulevarden 49, DK-2100 Copenhagen, Denmark

**Keywords:** microtubule-associated proteins, centrioles, basal bodies, cilia, ciliogenesis, axoneme, centrosome, microtubule inner proteins, microtubule outer proteins, mitotic spindle

## Abstract

**Highlights:**

**What are the main findings?**

- MAPs, MIPs, and MOPs act as central regulators of specialized microtubule architectures in centrioles, cilia, mitotic spindles, and neurons.
- Structural and comparative studies reveal that recurrent microtubule-binding modules are reused across compartments and adapted to distinct lattice geometries and functions.

**What is the implication of the main finding?**

- Specialized microtubule systems are built and maintained by modular protein networks, not by tubulin polymers alone.
- Disruption of these networks links microtubule architecture to ciliopathies, neurodevelopmental and neurodegenerative disorders, chromosomal instability, and cancer.

**Abstract:**

Microtubule-associated proteins (MAPs) are key regulators of microtubule architecture and dynamics, orchestrating microtubule stability, post-translational modification, and spatial organization across diverse cellular contexts. Through these activities, MAPs govern essential processes including cell division, intracellular transport, signaling, and differentiation. This review synthesizes current insights into how MAPs regulate a diverse range of cellular processes, including maintenance of structural integrity, centriole assembly, cell division, the dynamic transition between centrosomal and ciliary states, and neuronal growth and connectivity. We discuss advances from structural biology, proteomics, and cell imaging that are redefining the molecular landscape of centriole and cilia regulation, and we highlight emerging themes linking MAP dysfunction to human disease, including cancer, ciliopathies, and neurodegenerative disorders. By integrating these diverse perspectives, the review outlines a unifying framework for understanding how MAPs orchestrate microtubule function and identifies key challenges and opportunities for future research.

## 1. Background

The microtubule cytoskeleton, a dynamic filament system built from polymerized α/β-tubulin heterodimers, underlies a wide range of essential processes in eukaryotic cells [1]. Microtubules help determine cell architecture and intracellular organization [2], provide tracks for vesicle and organelle transport [3], drive mitotic chromosome segregation [4], contribute to cell polarity and migration [5], and form the structural core of cilia [6]. Cilia occur as either immotile (primary) or motile organelles. Primary cilia are usually solitary sensory organelles present on most vertebrate cells, whereas motile cilia are found on specialized epithelial cells where they generate fluid flow and the related sperm flagellum provides cellular propulsion [6]. These diverse functions require a balance between stability and plasticity. Microtubules must be robust enough to support cellular organization, while remaining capable of rapid remodeling in response to developmental, environmental, and cell-cycle cues. This balance is regulated not only by tubulin itself, but also by a diverse repertoire of microtubule-associated proteins (MAPs).

Structurally, microtubules are hollow cylindrical polymers composed of α/β-tubulin heterodimers arranged head-to-tail into polar protofilaments. Most cytoplasmic microtubules contain 13 protofilaments, although specialized structures such as centrioles and the axonemes of motile and primary cilia deviate from this canonical arrangement [1,7]. Tubulin polarity gives microtubules a more dynamic plus end and a generally more stable minus end [1,7,8].

Microtubule assembly is also GTP-dependent: GTP-bound β-tubulin favors growth, whereas GTP hydrolysis after lattice incorporation promotes instability. This cycle underlies dynamic instability, where individual microtubules switch between growth, shrinkage, catastrophe, and rescue [7,8]. The lattice is not uniform, as the seam forms a structural discontinuity with heterotypic lateral contacts and can be recognized or stabilized by specialized MAPs [7,9,10,11,12]. Microtubules are also chemically and compositionally diverse due to tubulin isotypes and post-translational modifications, which together contribute to the “tubulin code” and influence the recruitment and activity of motors, severing enzymes, plus-end factors, and structural MAPs [13,14].

**Figure 1:**
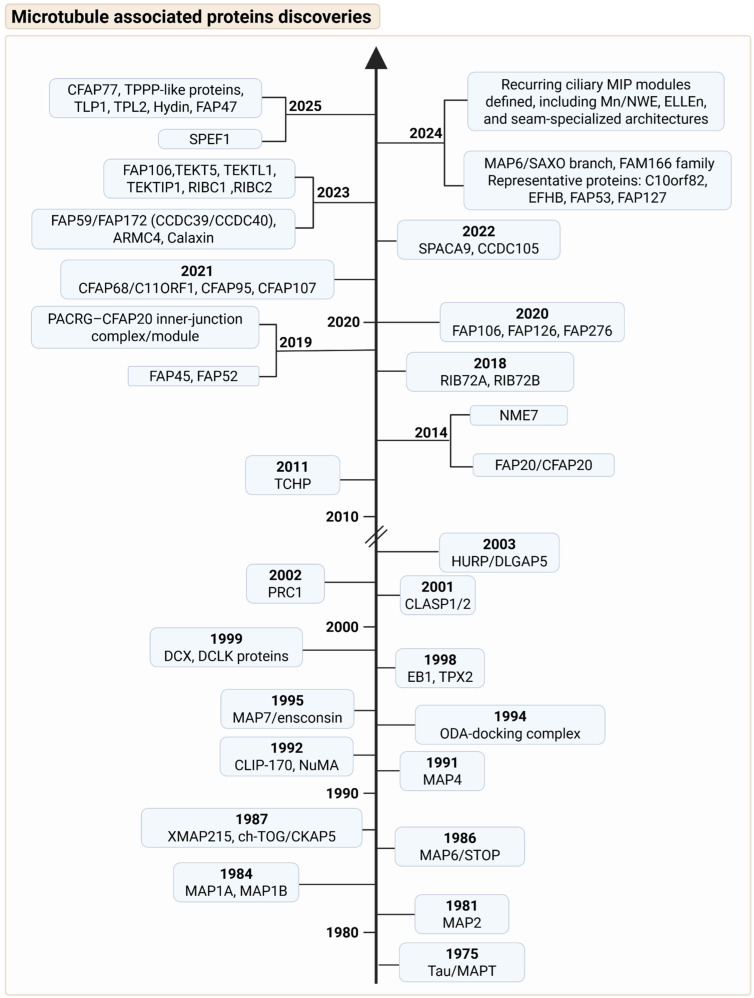
Timeline of representative microtubule-associated protein discoveries discussed in this review. The timeline summarizes selected microtubule-associated proteins (MAPs), microtubule inner proteins (MIPs), microtubule-organizing proteins (MOPs), protein complexes, families, and structural modules discussed throughout this review. Entries are arranged according to the approximate year in which each protein, complex, family, or structural module was first linked to a microtubule-associated structure or function relevant to the topic covered. Early discoveries include classical MAPs such as Tau/MAPT, MAP2, MAP1A/MAP1B, MAP6/STOP, MAP4, CLIP-170, and XMAP215/ch-TOG/CKAP5, whereas more recent studies increasingly define centriolar, ciliary, and axonemal MIPs, inner-junction complexes, molecular rulers, and recurring structural modules through advances in cryo-electron microscopy and proteomics. When multiple proteins or complexes are grouped within the same box, they indicate a similar historical period rather than simultaneous discovery or membership in a single molecular complex. The timeline is intended as a conceptual overview of representative milestones rather than a comprehensive chronology of all known microtubule-associated proteins.

This structural conservation combined with regulatory flexibility helps explain why microtubules depend on many classes of associated proteins. The concept of MAPs emerged from biochemical work in the 1970s [12,15] and has since expanded from classical lattice-binding stabilizers to a broad category that includes lattice binders, plus-end tracking proteins, nucleation factors, severing enzymes, tubulin-code regulators, motors, and adaptor proteins linking microtubules to membranes, actin networks, and signaling pathways (see Figure 1) [7,8,16]. To clarify this terminology, Table 1 summarizes the main categories of microtubule-associated proteins and related regulators and indicates which are the primary focus of this review.

**Figure 2:**
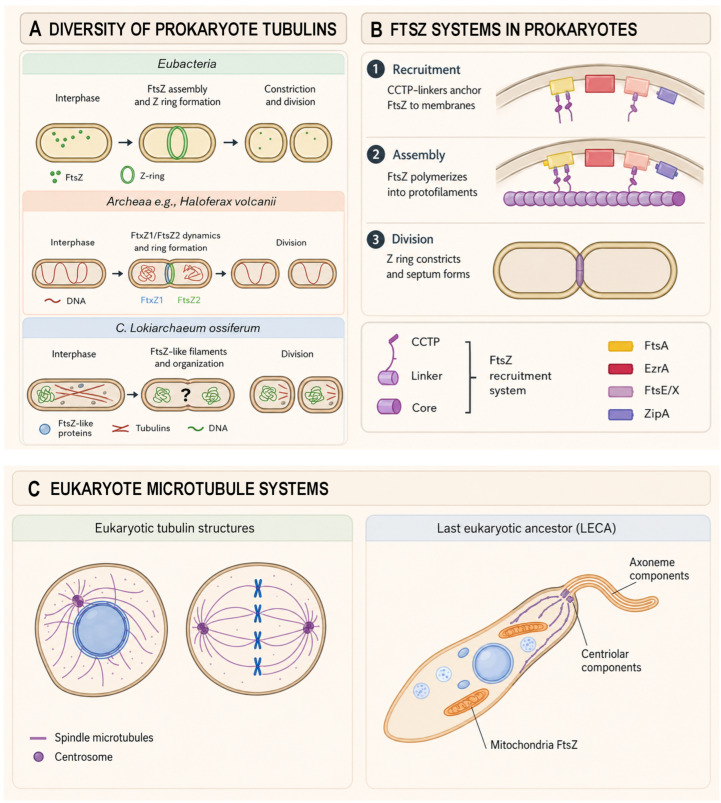
Diversity and evolution of tubulin systems across life. (**A**) Diversity of prokaryotic tubulins. In bacteria, FtsZ assembles into a midcell Z ring that drives cytokinesis. In Candidatus Lokiarchaeum ossiferum, Asgard tubulin homologs form α/β-tubulin heterodimers that can assemble into non-canonical microtubule-like polymers. (**B**) FtsZ systems in prokaryotes. FtsZ-mediated division proceeds through recruitment to the membrane via CCTP-dependent linkers, polymerization into protofilaments, and Z ring constriction to drive septum formation. (**C**) Eukaryotic microtubule systems. Eukaryotes evolved complex microtubule-based structures, including the mitotic spindle, centrosomes, centrioles, and ciliary axonemes. By the time of the last eukaryotic common ancestor (LECA), core features of this system were likely already present, including α/β-tubulin-based microtubules, centrioles or basal bodies, and axonemal structures.

Given the evolutionary roots of microtubules in prokaryotic tubulin-like proteins such as the filamenting temperature-sensitive mutant Z (FtsZ) [17,18], one might expect a highly conserved regulatory system. Instead, comparative studies show that MAP repertoires vary substantially across taxa [19], reflecting the diversification of microtubule functions from prokaryotic cytokinesis to specialized eukaryotic structures such as spindles, cilia, centrioles, and neuronal processes (Figure 2). In these systems, MAPs can be viewed as modulators of the stability–plasticity balance. This regulation is especially elaborate in centrioles and cilia, where microtubule inner proteins (MIPs), microtubule outer proteins (MOPs), and associated structural regulators stabilize unusual lattice geometries, recognize specific protofilament positions, and link microtubules to larger architectural assemblies [9,16].

Here, we comprehensively gauge the evolutionary and functional diversity of MAPs, examining how conserved microtubule-binding strategies have been adapted and expanded to support specialized microtubule systems and increasingly complex cellular environments. We focus particularly on MIPs, MOPs, and associated structural regulators that organize centrioles, cilia, mitotic spindles, neuronal microtubule arrays, and intracellular trafficking networks. The evolutionary diversification of these proteins is closely linked to major eukaryotic innovations, including the emergence of centrosomes, cilia, and higher-order microtubule architectures. Because many MAPs employ recurrent microtubule-binding domains (MBDs), motifs, and structural modules, Table 2 summarizes the principal binding strategies discussed throughout this review, together with their associated lattice features and representative proteins or complexes. The recent surge in the discovery of previously unrecognized MAPs and their modes of engagement with microtubules has revealed that MAP architectures recur across distinct microtubule compartments, suggesting that evolution has repeatedly repurposed common binding modules while acquiring new regulatory interfaces [9,16]. Tissue-specific expression, alternative splicing, and post-translational modification further aid these proteins, allowing the highly specialized microtubule systems to form [20,21,22]. We will therefore focus on the evolution of MAP microtubule-binding strategies, how and when these interfaces emerged, diversified, and adapted, and how their disruption contributes to disease. Through this lens, we will discuss how MAPs emerged not merely as auxiliary factors but as central architects of microtubule behavior, shaping the dynamic equilibrium that defines the eukaryotic cytoskeleton.

## 2. Evolution of MTs and MAPs

The evolutionary history of the microtubule cytoskeleton reflects a gradual elaboration of filament systems that predate the emergence of eukaryotic cells. The earliest recognizable antecedents of microtubules are found in bacteria, where the tubulin homolog FtsZ performs a central role in cytokinesis [18,23]. FtsZ polymerizes into protofilament-like structures that assemble into the Z-ring at the division site, guiding septum formation [24].

Although structurally simpler than eukaryotic microtubules and lacking the hollow cylindrical architecture, FtsZ filaments exhibit nucleotide-dependent polymerization dynamics and curvature transitions that foreshadow key aspects of tubulin behavior [23]. In this sense, FtsZ can be viewed as an ancestral scaffold upon which more elaborate filament systems were later constructed. Interestingly, although FtsZ sits at the center of a highly dynamic network in bacteria, mainly driving the formation of the Z-ring during cytokinesis, it engages only ~10–20 direct binding partners or regulators (https://pubmed.ncbi.nlm.nih.gov/28419603/ accessed on 13 July 2016, Figure 2) [25], of which just a small subset (~2–3) have been resolved in high-resolution co-structures [26]. Notably, none of these show clear orthology to the rich repertoire of MAPs found in eukaryotes.

This lack of detectable evolutionary continuity suggests that microtubule-associated regulatory systems in higher eukaryotes have largely arisen through convergent evolutionary processes. Although canonical MAPs are absent in bacteria and archaea, there are intriguing exceptions that hint at early MAP-like functionality. One notable example is *Bordetella pertussis*, the causative agent of whooping cough, which colonizes the ciliated respiratory epithelium of its human host. *Bordetella* species adhere to host respiratory cilia axonemes via the FhaB adhesin, which recently was shown to harbor a C-terminal MBD that directly engages axonemal microtubules [27]. The distribution of FtsZ-like proteins extends into archaea, where both canonical FtsZ and divergent tubulin-like proteins have been identified [28]. However, it is within the Asgard archaea, named after the deities of Norse mythology, to reflect their deep evolutionary significance, that a convincing evolutionary transition becomes apparent. In particular, a recent study of *Candidatus Lokiarchaeum ossiferum* (*C.L. ossiferum*) has revealed the presence of tubulin homologs that form heterodimeric complexes reminiscent of eukaryotic α/β-tubulin [29,30]. These proteins assemble into protofilament bundles and, under appropriate conditions, into tubular protofilament structures with a defined lumen, representing the earliest known instance of microtubule-like polymers outside eukaryotes. The emergence of such heterodimeric tubulin systems in *C.L. ossiferum* suggests that the fundamental architectural unit of microtubules, and the capacity for regulated protofilament organization, was already established before the rise of eukaryotic cellular complexity, although evidence for microtubule modulators awaits. However, the decisive transition to eukaryotic cells marks a profound expansion in both the complexity and diversity of microtubules and their associated proteins. By the time of the last eukaryotic common ancestor (LECA), the microtubule apparatus was already unexpectedly sophisticated, comprising α- and β-tubulin heterodimers together with γ-tubulin-dependent nucleation, and centriole/basal body–axoneme systems, many features of which are broadly conserved across major extant eukaryotic lineages [19] (Figure 2).

Moreover, LECA is thought to have possessed functional mitotic spindles and canonical axonemal structures (9 × 2 + 2), indicating that the key MAP networks for microtubule dynamics, division, and motility were already established at an early stage. Thus, rather than representing a primitive condition, the ancestral eukaryotic cell likely harbored a fully developed microtubule cytoskeleton. Insights into this early complexity are retained in modern unicellular eukaryotes such as *Naegleria gruberi*, *Chlamydomonas reinhardtii*, *Trypanosoma brucei*, and *Tetrahymena thermophila*, whose cellular organization reflects a wide range of specialized microtubule systems. The identification of many conserved MAP orthologs across these protists is consistent with an early diversification of microtubule-associated regulatory machinery [9]. Comparative studies indicate that the ancestral eukaryote possessed a flagellar apparatus with associated root microtubules that served as a central organizing framework for cell architecture, polarity, and motility. The widespread conservation of the axonemal structure (9 × 2 + 2) and basal body organization further supports the notion that these specialized arrays emerged early and became a defining feature of eukaryotic cells [31,32].

This evolutionary perspective suggests that the earliest MAPs were likely associated with these flagellar and basal body systems rather than with later-evolving cytoplasmic microtubule arrays such as in neurons. The inferred presence of complex axonemal and organizing structures in LECA implies that a diverse set of microtubule-binding and patterning proteins had already evolved, accompanying the early establishment of motility and intracellular organization. It is conceivable that the first MAPs were simple tubulin-binding peptides or domains that stabilized protofilament interactions or modulated nucleotide-dependent dynamics. Over evolutionary time, these primitive elements may have diversified into the distinct functional classes observed today, including stabilizers, destabilizers, motors, and plus-end tracking proteins. The absence of clear homologs between prokaryotic regulators and eukaryotic MAPs suggests either rapid divergence or independent innovation driven by similar selective pressures. A further layer of complexity arises from the spatial organization of MAP interactions with the microtubule lattice. Broadly, these can be divided into microtubule inner proteins (MIPs), which localize within the lumen, and microtubule outer proteins (MOPs), which bind to the external surface. MIPs are particularly prominent in stable microtubule structures such as centrioles and cilia/flagella, where they contribute to lattice integrity and mechanical resilience [9,10]. Among the MIPs and MOPs, proteins associated with the microtubule seam, a unique structural discontinuity in the lattice, have attracted particular attention [9,10,11,33]. The seam represents a region where lateral contacts between protofilaments differ from the canonical arrangement, potentially serving as a site for regulatory interactions. Protein families such as CFAP68/C11ORF1, CFAP95, CFAP107, SPAG8, and SPEF1 have been implicated in recognizing and stabilizing this region either from within or on the outside of microtubules [9,10,11], suggesting that even subtle structural features of the microtubule lattice have been exploited during evolution to achieve regulatory specificity.

Taken together, the evolution of microtubules and MAPs reflects a continuum from simple, self-organizing filament systems to highly regulated and functionally diverse cytoskeletal networks. The emergence of heterodimeric tubulin and tubular polymers in Asgard archaea provides a critical link in this trajectory, while the expansion of MAP repertoires in eukaryotes underscores the importance of regulatory innovation. The interplay between intrinsic filament properties and extrinsic modulators continues to define the behavior of microtubules, echoing an evolutionary history shaped by the dual demands of stability and adaptability.

## 3. Writing, Erasing, and Reading the Tubulin Code: MAPs as Interpreters of Microtubule Identity

**Figure 3:**
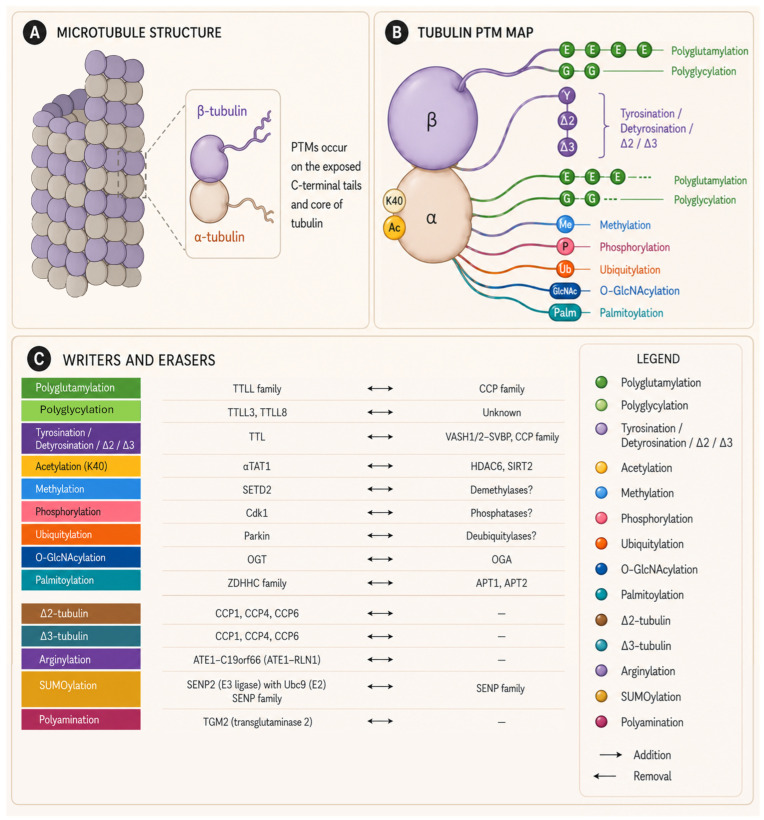
Microtubule structure and post-translational modifications (PTMs). (**A**) Microtubule structure. Microtubules are composed of α/β-tubulin heterodimers arranged head-to-tail into protofilaments that form a hollow cylindrical lattice. Tubulin PTMs occur mainly on the exposed C-terminal tails, although some modifications are located within the tubulin core or lumen-facing regions. (**B**) Tubulin PTM map. Schematic overview of major tubulin PTMs. The α-tubulin C-terminal tail is subject to the tyrosination–detyrosination cycle and subsequent generation of Δ2- and Δ3-tubulin, while both α- and β-tubulin C-terminal tails can undergo polyglutamylation and polyglycylation. α-Tubulin is also modified by acetylation at K40, a lumen-facing residue, and additional PTMs include methylation, phosphorylation, ubiquitylation, O-Linked β-N-Acetylglucosamine (O-GlcNAcylation), and palmitoylation. (**C**) Writers and erasers. Enzymes responsible for the addition (“writers”) and removal (“erasers”) of tubulin PTMs. Tyrosination is catalyzed by TTL, whereas detyrosination is mediated by the VASH1/2–SVBP complex. Polyglutamylation and polyglycylation are catalyzed by TTLL family enzymes and reversed by CCP family members where known. α-Tubulin K40 acetylation is mediated by αTAT1 and reversed mainly by HDAC6 and SIRT2. The figure also summarizes the currently known enzymes involved in Δ2- and Δ3-tubulin formation, arginylation, SUMOylation, and polyamination, where these have been experimentally established. Additional PTMs are mediated by enzymes such as SETD2, Cdk1, Parkin, and OGT/OGA, although the corresponding writer and eraser systems remain incompletely defined for several tubulin modifications.

The concept of a tubulin code emerged from the realization that microtubules, despite their conserved polymer architecture, are not chemically uniform. Rather, they are diversified by the combined action of tubulin isotypes and post-translational modifications, which together generate biochemically distinct microtubule subsets that can be differentially interpreted by associated factors. In its mature formulation, the tubulin code is thus not merely a catalog of modifications, but an information system written onto the tubulin surface and lumen, and decoded by motors, severing enzymes, plus-end factors, and structural MAPs. This principle has been articulated most clearly in the landmark syntheses of Janke & Magiera, and Roll-Mecak and colleagues [13,14], which established the conceptual framework that still governs the field. At the center of this code are a limited number of major “writer” enzyme families, of which we here highlight the best characterized (Figure 3). The re-tyrosination reaction is catalyzed by tubulin tyrosine ligase (TTL), the founding member of the TTL-like superfamily. TTL acts on detyrosinated α-tubulin and restores the genetically encoded C-terminal tyrosine, thereby regenerating the tyrosinated state that characterizes younger and more dynamic microtubules [34]. Structural studies showed that TTL recognizes the curved α/β-tubulin dimer rather than the straight microtubule lattice, explaining why re-tyrosination occurs primarily on soluble tubulin before polymer incorporation [35,36]. These studies also established the conserved TTL fold as the structural basis for the broader TTLL family.

Different TTLL enzymes show interesting substrate preferences. Some initiate branch formation, whereas others elongate existing chains. Some prefer α-tubulin, others β-tubulin, and some act predominantly on soluble tubulin, whereas others recognize the microtubule lattice. Biochemical and structural work on TTLL7, TTLL6, TTLL3 [37,38,39,40], and, more recently, TTLL11 [41] has shown that this specificity is encoded in distinct modes of tail capture and lattice engagement built upon the shared TTL-like catalytic core [37,38,39,40,41]. Particularly important in this respect are the cryo-EM and biochemical studies showing that TTLL enzymes can read both tubulin subunit identity and prior modification state, thereby creating combinatorial hierarchies within the code itself.

A second major writer system is represented by the glutamylases and glycylases of the TTLL family. These enzymes modify glutamate residues within the unstructured C-terminal tails of α- and β-tubulin by adding either glutamate or glycine side chains [41,42]. For glutamylation, erasure is carried out by the cytosolic carboxypeptidases (CCPs), including CCP1, CCP2, CCP3, CCP5, and CCP6. These enzymes do not all perform the same reaction: CCP1, CCP2, and CCP3 shorten side chains, whereas CCP5 is unusual in being able to remove the branch-point γ-linked glutamate itself [43,44,45]. Genetic, biochemical, and disease studies established that loss of CCP activity perturbs cilia, axons, and neuronal survival, underscoring that the code depends not simply on deposition of marks, but on the maintenance of homeostatic modification levels.

Glycylation, by contrast, remains the least fully decoded major branch of the tubulin code. In mammals, glycylation is initiated mainly by TTLL3 and TTLL8, while TTLL10 has been implicated in elongation or context-dependent polyglycylation. This modification is especially enriched in axonemal microtubules [42]. Genetic studies in ciliates and vertebrates have established that glycylation is crucial for ciliary and flagellar function [46,47], and more recent work has shown that it can modulate outer-arm dynein behavior and occupy spatially restricted protofilament patterns in motile cilia [48,49,50]. Yet, compared with tyrosination and glutamylation, the reader mechanisms for glycylation remain less well resolved at the level of individual domains. Here, the field is still transitioning from phenomenology to mechanism. One of the most important lessons from the literature is that not all marks are read in the same way. Tyrosination creates a discrete terminal epitope that can be recognized by a defined domain. Glutamylation and glycylation alter the electrostatic and steric properties of the tubulin tails, thereby tuning the activity of proteins that engage these flexible regions. Acetylation, being luminal, often acts less as a canonical recruitment signal than as a regulator of lattice mechanics and damage tolerance. Accordingly, the tubulin code should not be imagined as a single mode of molecular recognition, but as a layered system in which some marks recruit, others tune, and still others alter the physical state of the polymer itself.

Acetylation constitutes a mechanistically distinct branch of the code. The principal writer of α-tubulin K40 acetylation is αTAT1, also known as ATAT1, which is necessary and largely sufficient for this modification in mammals [51,52,53]. Unlike most tubulin PTMs, K40 acetylation is located on the luminal side of the microtubule, and this unusual topology is mirrored by the enzyme mechanism [54,55]. Structural studies showed that αTAT1 is a Gcn5-related acetyltransferase with a relatively open substrate-binding groove [56], while mechanistic work demonstrated that the enzyme gains access to K40 through microtubule ends and lattice defects. Subsequent studies refined the picture further by showing that acetylation does not primarily create a classic external docking epitope; rather, it alters the conformational landscape of the K40 loop and increases lattice resilience to mechanical stress [57]. The deacetylation branch is dominated by HDAC6 and, in some contexts, SIRT2. HDAC6 was first identified as a tubulin deacetylase more than two decades ago [58], and later biochemical work showed that it has a strong preference for free tubulin dimers over the assembled microtubule lattice [59], a finding that helps explain the kinetic asymmetry of the acetylation cycle. SIRT2 can also contribute to α-tubulin deacetylation in cells [60], although HDAC6 remains the principal and best-established deacetylase in most experimental systems.

The code is equally dependent on erasers. In the tyrosination cycle, the major detyrosinating enzymes are the vasohibins, especially VASH1 and VASH2, which require the small binding partner SVBP for full activity and stability. Structural studies of the VASH1–SVBP complex revealed that vasohibins belong to a transglutaminase-like cysteine protease fold and recognize the acidic α-tubulin tail through an extended groove positioned to cleave the terminal tyrosine [61,62]. These studies provided the first direct structural account of detyrosination and explained how detyrosinating enzymes engage the microtubule surface. The functional significance of detyrosination is particularly evident in mitosis, where it stabilizes kinetochore–microtubule attachments and biases error correction, thereby directly influencing chromosome segregation fidelity and genomic stability [63]. If writers and erasers define the alphabet of the code, the readers determine its meaning. The clearest and best-validated reader module is the Cytoskeleton-associated protein glycine-rich domain (CAP-Gly) domain. CAP-Gly domains in proteins such as p150^Glued^, CLIP-170, and KIF13B recognize the C-terminal glutamate–glutamate–tyrosine/phenylalanine (EEY/F) motif present on tyrosinated α-tubulin [64,65,66]. Structural and cell biological studies showed that this interaction depends on a conserved GKNDG-containing loop within the CAP-Gly fold and is strongly reduced upon detyrosination [64,65]. In this manner, tyrosination promotes the recruitment of CAP-Gly proteins to microtubule plus ends and thereby influences the initiation of dynein-driven transport [65]. The CAP-Gly–EEY interaction remains the paradigmatic example of direct tubulin-code readout by a defined protein domain.

Detyrosination is also read by motors, although in this case the readout appears more distributed than in the CAP-Gly system. Classical work in neurons showed that kinesin-1 preferentially engages detyrosinated microtubules for polarized transport, thereby linking the tyrosination state of tubulin to axonal identity [67]. Yet purified-motor reconstitution experiments indicate that the effect of detyrosination on kinesin motility is modest in isolation, implying that in cells it is likely integrated with other lattice features, MAPs, and regional microtubule age [68]. Thus, detyrosination is best viewed as a contextual signal rather than a simple on–off docking site for kinesin.

The most striking reader of polyglutamylation is the severing enzyme spastin. Landmark work showed that long glutamate side chains strongly stimulate spastin-mediated severing, and later reconstitution experiments demonstrated that glutamylation acts as a rheostat: increasing severing over a substantial range, yet eventually becoming inhibitory when chain length becomes excessive [69]. Structural studies showed that spastin can engage glutamate-rich tubulin tails through a positively charged substrate-binding surface, suggesting a mechanistic basis for how tail chemistry may influence the AAA ATPase severing machinery [70,71]. In this way, polyglutamylation is not merely correlated with severing but directly controls it. Polyglutamylation also tunes the interaction of microtubules with other effectors, including motors and MAPs, especially in axons and cilia. More recent biochemical work has emphasized that distinct glutamylases generate chemically and functionally distinct patterns, and that α- and β-tubulin glutamylation are not equivalent signals. This refinement is important, because it suggests that the code is not read only at the level of “modified versus unmodified,” but also at the level of chain length, branch architecture, tubulin subunit identity, and protofilament position.

Taken together, the prominent studies in the field support a view of the tubulin code as a chemically elaborate and structurally stratified regulatory language. It is written by TTL, TTLL glutamylases and glycylases, and αTAT1; erased by vasohibins, CCPs, HDAC6, and SIRT2; and read by modules such as CAP-Gly domains, by severing enzymes such as spastin, and by motors whose preferences are shaped by the age and chemical state of the lattice. What has emerged from these studies is not simply that microtubules are modified, but that their modifications are organized, interpreted, and biologically consequential. The code, in other words, resides not in any single mark, but in the evolving relationship between tubulin chemistry and the proteins that have learned to read it.

## 4. MAPs Associated with Complex Microtubule Structures

### 4.1. MAPs of Centrioles and Basal Bodies

**Figure 4:**
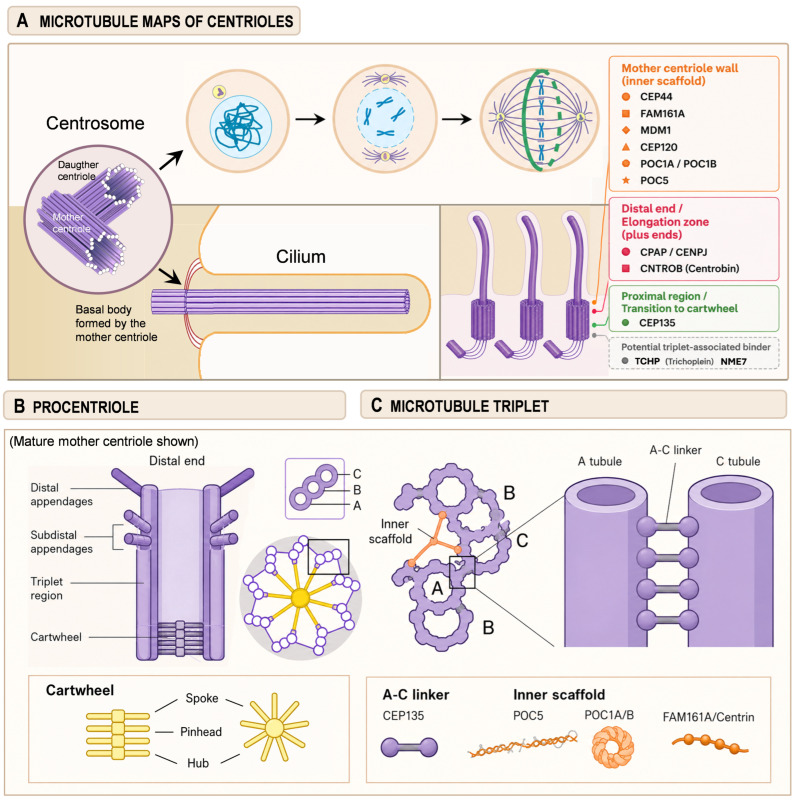
Microtubule organization and architecture of centrioles and cilia. (**A**) Microtubule MAPs of centrioles. Centrosomes serve as major microtubule-organizing centers that nucleate spindle microtubules during mitosis. In interphase, centrioles transition into basal bodies that template the formation of cilia. The basal body anchors the axoneme, which extends to form the cilium, highlighting the dual role of centrioles in cell division and primary cilium growth, typically based on a (9 × 2 + 0) axoneme. (**B**) Centriole structure. Schematic of a centriole showing its proximal–distal polarity and characteristic ninefold symmetry. The proximal region contains the cartwheel structure, composed of a central hub and radial spokes that establish ninefold symmetry, while the distal end is associated with appendages and microtubule extensions. (**C**) Microtubule triplet organization. Centrioles have a (9 × 3 + 0) organization, consisting of nine A-, B-, and C-tubule triplets and no central pair. Key structural components include CEP135 (A–C linker), POC1A/B, FAM161A, Centrin, and POC5 (inner scaffold), which support centriole assembly and integrity.

The previous section outlined how MAPs have evolved by diversifying a relatively small set of microtubule-binding strategies. In cilia, these strategies are used in a highly position-specific way to stabilize the axoneme (as discussed later). This perspective also helps frame centrioles, where similar binding principles are adapted to the distinct (9 × 3 + 0) triplet-microtubule geometry. The distinction between centrioles and cilia is real. Centrioles are not merely remnants of cilia that were repurposed for mitosis but represent a structurally and functionally distinct cellular compartment (Figure 4). This distinction extends to the level of gene regulation circuits, where centriole- and cilia-associated proteins are governed by partially divergent transcriptional programs [72]. While structurally highly intertwined in the cell and many centriole components co-evolved with ciliary proteins, reflecting their shared structural ancestry [31,32], the centriole and cilia patterns of gene co-expression reveal clear compartment-specific regulation. Ciliogenesis is controlled by transcriptional programs including RFX factors, while FOXJ1 is particularly important for motile cilia gene expression [73,74]. In contrast, centriole biogenesis is closely linked to cell cycle machinery, with key regulators including E2F transcription factors and PLK4-associated pathways, which coordinate centriole duplication with DNA replication [75,76]. Taken together, these observations suggest that, although centrioles and basal bodies are evolutionarily related, the mechanisms that govern centriole and basal body biology and those that govern ciliary function are maintained by partly overlapping yet distinct regulatory programs adapted to their specific cellular roles.

Centrioles are conserved (9 × 3 + 0) microtubule-based cylinders that duplicate once per cell cycle and organize both centrosomes and cilia [77,78]. In proliferating cells, paired centrioles recruit pericentriolar material to form the centrosome, a major microtubule-organizing center important for cytoplasmic microtubule organization and mitotic spindle assembly [79]. Upon maturation, the mother centriole acquires distal and subdistal appendages: distal appendages support membrane docking and ciliogenesis, whereas subdistal appendages anchor microtubules [80]. During ciliogenesis, the mother centriole converts into a basal body, docks to the membrane, and templates axoneme extension [78,80]. Thus, proteins controlling centriole structure often also affect cilium assembly and stability [77,78].

Centriole architecture is critically defined by the cartwheel structure, which establishes the ninefold (9 × 3 + 0) organization and connects the microtubule triplets at early stages of centriole assembly (Figure 4). The cartwheel is built around the central hub protein SAS-6 [81], which oligomerizes into a ring-like structure from which radial spokes extend toward the A-tubules of each triplet. Structural studies showed that lateral interactions between SAS-6 dimers impose an angle of approximately 40°, providing a physical basis for ninefold symmetry, as nine 40° units complete a 360° ring [82,83]. These spokes are linked to the microtubule wall through proteins such as CEP135 (Bld10) and STIL, which stabilize the connection between the cartwheel and the triplet microtubules [84,85]. Additional components, including CPAP (CENPJ), contribute to microtubule elongation and may link the cartwheel to the growing microtubule wall [84], while proteins such as CEP120 and SPICE1 further support triplet assembly and stability [86,87]. Additional factors such as RTTN and CEP295 contribute to centriole-to-microtubule coupling and maturation [88,89], further reinforcing the connection between the cartwheel and the triplet wall. Although not all cartwheel components directly bind tubulin with equal affinity, together they form an architecture that physically couples the central hub to the microtubule triplets, thereby acting as an essential organizer of centriole microtubule architecture (Figure 4) [90]. Cryo-electron tomography studies including human centrioles have visualized the cartwheel region, but high-resolution molecular detail is still largely derived from non-human systems [81,91,92,93]. Beyond the cartwheel and inner scaffold, centriole architecture is further stabilized and functionally specialized by additional microtubule-associated structures, including the recently resolved A–C linker and the proximal and distal appendages. The A–C linker physically connects the A-tubule of one triplet microtubule to the C-tubule of the adjacent triplet, forming a circumferential network that reinforces ninefold symmetry and mechanical integrity [94,95,96]. Recent cryo-EM studies have revealed that this linker is composed of a defined set of proteins that directly interface with the microtubule wall, thereby acting as inter-triplet MAPs that stabilize the outer architecture of the centriole. In parallel, centriole appendages represent specialized outer MAP assemblies with distinct positional and functional roles. Centrosome cohesion is mediated primarily by a proximal linker that connects duplicated centrosomes, whereas mother centriole appendages comprise functionally distinct subdistal and distal appendages. Subdistal appendages contribute to centrosomal microtubule anchoring and organization, while distal appendages (including CEP83, SCLT1, and CEP164) mediate membrane docking and are essential for ciliogenesis [97,98,99]. Although not all appendage components are direct tubulin binders, these outer centriole-associated assemblies functionally link centriolar microtubules to membranes and signaling machinery. Together with inner scaffold proteins and triplet MIPs, these structures highlight that centriole integrity relies on a multi-layered system of MAPs that includes internal reinforcement, inter-triplet linkers, and external functional appendages.

While outer structures such as the A–C linker and appendages stabilize the centriole externally, maintenance of triplet integrity along its length depends on internal scaffold systems. A key component of this inner architecture is the MAP FAM161A, which links microtubule structure to long-range stability. In human cells, FAM161A forms a complex with POC5, POC1B, Centrin-2, and CCDC15, but the microtubule-binding activity in that complex is attributed primarily to FAM161A [100,101]. This inner scaffold maintains triplet cohesion and centriole integrity, conceptually mirroring the connecting-cilium inner scaffold in photoreceptors [102]. Notably, classic in vitro work showed that centrioles can retain their overall configuration even after experimental disassembly of centriolar microtubules by high-salt treatment, consistent with the idea that a non-microtubule scaffold contributes to structural persistence [103]. A second direct centriolar microtubule binder is WDR90/POC16, which was shown to localize on the centriolar microtubule wall and to bind both tubulin and microtubules directly. WDR90 seems to connect the inner scaffold to the triplet wall, so it functions less like an internal strut and more like an anchor between the scaffold and the tubulin shell [104]. A third important direct binder is HYLS1, which promotes centriole triplet microtubule assembly by engaging the β-tubulin C-terminal tail. A recent study places HYLS1 as a regulator of the unusual incomplete microtubules that define the triplet architecture [105]. This gives HYLS1 a different mechanistic role from FAM161A or WDR90. Rather than mainly stabilizing a finished wall, it helps make the triplet geometry possible in the first place [105].

Recent cryo-EM studies further indicate that centriole microtubule triplets are reinforced by MIPs lining the luminal surface of the A-, B-, and C-tubules. Although many of these remain unassigned, they are thought to stabilize the unique geometry of triplet microtubules, highlighting that centriole integrity depends on both external scaffold proteins and an internal MIP network.

### 4.2. MAPs in Human Cilia

Upon membrane docking, a centriole can transition into a basal body that serves as a template for cilium formation, thereby linking centriole architecture to axonemal microtubule organization. Following this transition, the (9 × 3 + 0) triplet microtubule architecture of the centriole gives rise to the doublet-based axoneme of cilia, typically (9 × 2 + 0) in primary cilia and (9 × 2 + 2) in motile cilia [106,107]. Here, an expanded repertoire of MAPs, including both MIPs and MOPs, further refines microtubule structure and function. Notably, ciliary MIPs tend to display strong evolutionary conservation, which can be rationalized by their use of recurrent, often repetitive microtubule-binding modules, as outlined below. These interact directly with the luminal surface of tubulin in a geometry-constrained manner, imposing strict structural requirements that limit sequence divergence. In contrast, ciliary MOPs generally lack such repetitive, lattice-imprinted binding modules and instead rely on more variable, often multidomain architectures to mediate interactions with motors, regulatory complexes, and membranes [108,109,110,111,112,113,114]. As a result, MOPs exhibit greater evolutionary plasticity, with many showing reduced sequence conservation and, in some cases, only becoming apparent through structural approaches rather than sequence based. One major tubulin-recognition structure is the MAP6/SAXO-type Mn module (Mn) repeat family of the classical MAP6/SAXO branch and several sperm-enriched paralogues [9,10,110,113,114]. Mn units generally bind a tubulin heterodimer from the luminal side and are often repeated in tandem, producing longitudinal arrays that are thought to reinforce the inner wall. A closely related specialization is the Asparagine, Tryptophan, Glutamic acid (NWE) module, a seam-specialized triad built from two Mn-like units paired with an N-terminal seam-binding NWE motif [9]. These proteins preferentially decorate the A-tubule seam, making contacts across the heterotypic lattice and thus appearing optimized for the structurally weak seam region [9]. This nicely illustrates how one ancestral tubulin-binding logic diversified into a general lattice binder and a seam-specialized binder. A second solution is the use of short tandem repeat modules. Proline–tyrosine–glycine (PYG) repeats form loop-like elements that contact adjacent tubulins through conserved proline/tyrosine-containing motifs, whereas glycine–phenylalanine–glycine (GFG) repeats use a conserved glycine–phenylalanine–glycine signature to engage the luminal lattice near the seam [9]. Human representatives include the FAM166 family and C10orf82 for PYG, and EFHB plus CFAP77 for GFG. Together, these families argue that ciliary MAP evolution repeatedly converged on short, modular helices or loops that can be repeated, mixed, or positioned to match different protofilament environments inside the doublet.

#### 4.2.1. MAPs of the Cilia Central Pair

**Figure 5:**
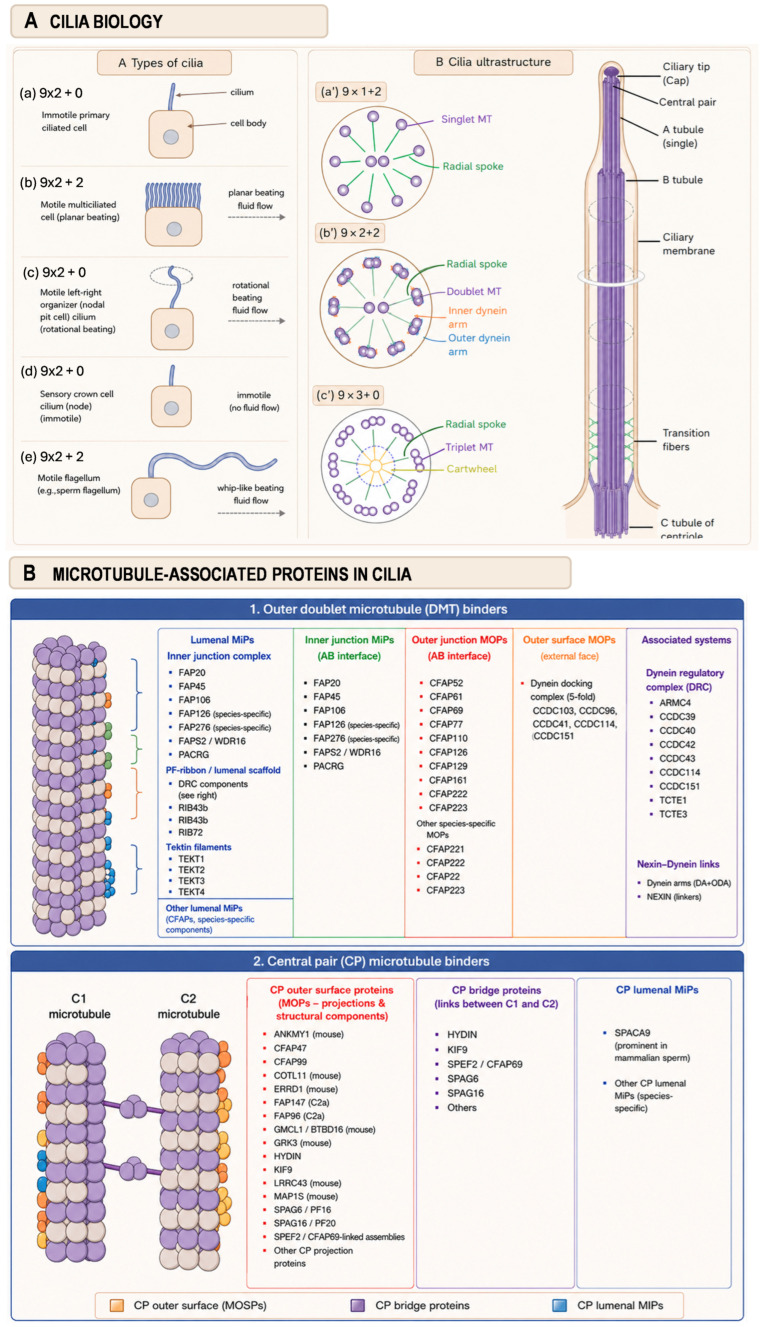
Cilium ultrastructure and MAPs in cilia. (**A**) Cilium ultrastructure. Left: Different types of cilia, including immotile primary cilia (9 × 2 + 0), motile multiciliated cells generating planar fluid flow (9 × 2 + 2), motile rotational cilia involved in left–right patterning (9 × 2 + 0), nodal cilia (9 × 2 + 0), and motile flagella (9 × 2 + 2). Right: Cross-sectional organization of ciliary axonemes. The canonical (9 × 2 + 2) structure contains nine outer microtubule doublets surrounding a central pair, with associated radial spokes and inner and outer dynein arms. Variants include 9 × 2 + 0 arrangements lacking the central pair. The axoneme is enclosed by the ciliary membrane and anchored at the basal body. For illustrative purposes, the schematic depicts the cartwheel structure within the basal body. In vertebrates, however, the cartwheel is largely or completely disassembled during basal body maturation and is absent from mature motile cilia, whereas it is retained or only partially reduced in several other organisms, including Chlamydomonas, protozoa, and insects. (**B**) Microtubule binders in cilia. Comprehensive overview of proteins associated with axonemal microtubules. Outer doublet microtubules (DMTs) are decorated by luminal microtubule inner proteins (MIPs), inner and outer junction proteins at the A–B interface, and microtubule outer proteins (MOPs), including dynein arms and regulatory complexes such as the dynein regulatory complex (DRC) and nexin–dynein links. The central pair (CP) microtubules (**C1**,**C2**) harbor distinct sets of outer surface projections, bridge proteins linking the two singlets, and luminal MIPs. Together, these components coordinate axonemal structure, stability, and motility.

Recent cryo-EM and cryo-ET studies have fundamentally changed our understanding of the central pair (CP) microtubules in motile (9 × 2 + 2) cilia [11,115]. Rather than being relatively simple singlet microtubules, the central pair (CP) is now recognized as a densely decorated, highly specialized microtubule system composed of dozens of proteins that bind both the outer surface and lumen, with strong positional specificity along the lattice [11,113,115]. A key conceptual advance is that CP MAPs display distinct and recurrent binding modes, many of which parallel those observed in doublet MIPs but are adapted to singlet microtubule geometry and the regulatory role of the CP. The CP apparatus of motile (9 × 2 + 2) cilia has long been viewed primarily as a regulatory structure, but recent cryo-electron microscopy and tomography studies have revealed that its microtubules are extensively patterned by a diverse and highly ordered set of MAPs [115,116]. These proteins bind directly to the microtubule lattice, both externally and internally, and display striking positional specificity. Rather than representing a simplified microtubule system, the CP emerges as a densely decorated and functionally specialized singlet microtubule pair, in which MAPs encode both mechanical stability and regulatory output (Figure 5). A major conceptual advance from recent structural work is that CP MAPs can be grouped according to distinct binding modes that read specific features of the tubulin lattice, including the seam, local curvature, and protofilament identity. These binding strategies parallel those observed in doublet microtubules but are adapted to the unique geometry and regulatory role of the CP [11,113,115]. One of the most significant findings is the identification of seam-specific MAPs within the CP.

#### 4.2.2. Outer-Surface MAPs Are Less Motif-Defined but Follow the Same Logic of Positional Specialization

Cryo-EM studies have revealed a diverse and extensive set of ciliary microtubule-associated proteins (CIMAPs) on the outer surface of doublet microtubules and central pair [117]. Some of these appear to be ubiquitous but cell-type-tuned. For example, CIMAP3 is shared broadly, whereas CIMAP2 appears sperm-specific, and sperm additionally carry external MAPs such as SPMAP1/2, CFAP97D1, EFCAB3 and TSSK-associated structures [114,115,118]. Even where motif-level assignments are still incomplete, the theme is the same as for luminal MIPs. Binding is highly position-specific, often to a particular protofilament cleft or to a site immediately adjacent to another axonemal complex. In other words, outer-surface MAPs are not generic decorations. They are local mechanical or regulatory couplers embedded at defined addresses on the doublet wall. For the photoreceptor connecting cilium, the best-supported directly microtubule-associated scaffold is the connecting-cilium inner scaffold, composed principally of FAM161A, POC5 and centrin, as for centriole microtubule triplets [100,119], which runs along the inner wall of the microtubule doublets and behaves like a “structural zipper” that keeps the doublets cohesive [120]. Recent studies have shown that this scaffold is essential for doublet cohesion and that loss of FAM161A abolishes the scaffold and leads to doublet spreading and retinal degeneration [120,121,122]. But the photoreceptor field still does not yet offer the same near-atomic census of directly tubulin-contacting MAPs that is now available for mammalian motile axonemes.

#### 4.2.3. Tektins and Sperm-Specialized MIPs Provide a Second Layer of Reinforcement

Across mammalian motile cilia, the most conspicuous non-repeat lumenal system is the tektin bundle. Tektins are long coiled-coil filaments positioned within the doublet lumen and are conserved across epithelial motile cilia and sperm, although sperm carry the most elaborate version [123], including TEKT1-5, TEKTL1, TEKTIP1 and RIBC1/2 [111]. These filaments are structurally distinct from the short-repeat MIPs: instead of reading local lattice geometry with small motifs, they form extended polymers that likely act as internal struts. Sperm add still more direct binders, including TEKT5, CCDC105 and SPACA9, and broader structural analyses of sperm DMTs identified many sperm-specific MIPs, including FAM166-family proteins and other sperm MIPs, consistent with the need to stiffen and tune the exceptionally long and mechanically stressed sperm axoneme [112,123].

The protein SPEF1 binds directly at the α/β-tubulin seam, contacting both subunits across the heterotypic interface. This is notable because the seam represents a structurally discontinuous and mechanically vulnerable region of the microtubule. By selectively targeting this site, SPEF1 stabilizes the lattice and, importantly, is also capable of crosslinking adjacent microtubules [11,124,125]. This establishes seam recognition as a dedicated microtubule-binding strategy within cilia, analogous to seam-targeting MIPs in doublet microtubules, but here implemented on singlet microtubules with additional crosslinking capacity.

A second major class of CP MAPs is defined by curvature-sensitive binding, exemplified by proteins containing TPPP-like domains [11,126,127]. These proteins, including TPPP-like proteins such as TLP1 and TLP2, bind along the outer surface of CP microtubules and appear to preferentially associate with regions of non-canonical lattice geometry, such as the curved microtubules found at the ciliary tip. Structural analyses show that these proteins can wrap laterally around the microtubule and, in some cases, bridge between the two central microtubules [11]. This suggests that TPPP-like domains function as curvature-sensing modules, stabilizing stressed or bent lattice conformations and contributing to the integrity of the CP under mechanical load. The ability of these proteins to span microtubules further indicates a dual role in both local lattice stabilization and higher-order organization. Beyond seam recognition and curvature sensing, the CP is characterized by an extensive network of crosslinking MAPs that connect the two central microtubules (C1 and C2) [11]. These proteins form periodic bridges with defined spacing, maintaining the precise alignment and distance between the microtubules. While some of these crosslinks are mediated by TPPP-domain proteins, additional components contribute to a highly regular internal architecture [113]. Functionally, these crosslinkers are likely critical for preserving CP geometry during ciliary bending and may also facilitate transmission of mechanical or regulatory signals between the two microtubules. In this respect, they resemble structural systems such as tektin bundles in doublet microtubules and inner scaffold components of centrioles [114], highlighting a conserved architectural principle of microtubule crosslinking in specialized arrays. The outer surface of CP microtubules is further decorated by periodic arrays of MAPs that bind to specific protofilaments with strict register (Figure 5). These proteins form large projection complexes that extend outward from the microtubule surface and interact with radial spokes. Importantly, many of these projections are asymmetric between the C1 and C2 microtubules, establishing functional polarity within the CP. Although the detailed domain architecture of all projection proteins is not yet fully resolved, the identified conserved modules within this repertoire, include proteins such as Hydin and FAP47, which belong to the ASPM-SPD-2-Hydin/major sperm protein ASH/MSP domain family [128,129,130,131]. Earlier bioinformatic analyses had suggested that ASH domains are associated with microtubule-based structures [128], but direct evidence for microtubule binding was lacking. In contrast, these structural studies now place ASH-domain-containing proteins directly on the microtubule lattice, providing the first convincing evidence that this domain family can mediate microtubule binding. Together with additional conserved elements, including TPPP-like domains, these findings indicate that CP projections comprise a diverse class of outer-surface regulatory MAPs. Through their connections to radial spokes, these proteins play a central role in coordinating dynein activity and, consequently, in defining the waveform and beat pattern of motile cilia [113,115]. In addition to external MAPs, accumulating evidence supports the presence of lumenal MIPs within CP microtubules [113,115]. These proteins bind the inner wall of the microtubule and contribute to lattice stabilization from within. Although less well characterized than their counterparts in doublet microtubules, CP MIPs appear to form a structured internal network that enhances rigidity and resistance to deformation. The presence of both lumenal and external MAP systems reinforces the view that CP microtubules, like doublets, are composite structures stabilized by coordinated inner and outer protein assemblies. A defining feature of CP MAP organization is its strong link to function. Unlike many doublet MAPs, which primarily reinforce structural integrity, CP MAPs are intimately connected to the regulation of motility. The asymmetric distribution of MAPs between C1 and C2, their periodic arrangement, and their interactions with radial spokes collectively establish a platform for controlling dynein activity. Thus, CP MAPs integrate structural stabilization with regulatory signaling, ensuring that mechanical forces generated by dyneins are properly coordinated across the axoneme.

Taken together, recent cryo-EM studies reveal that the central pair is not merely a passive structural element but a highly specialized microtubule system patterned by multiple classes of MAPs. These include seam-binding proteins such as SPEF1, curvature-sensitive TPPP-domain proteins, crosslinking MAPs that bridge the two microtubules, outer-surface projection complexes, and lumenal MIPs. Each class reads distinct features of the microtubule lattice and contributes to a unified architecture that supports both stability and regulation.

### 4.3. Mitotic Spindle-Associated MAPs

**Figure 6:**
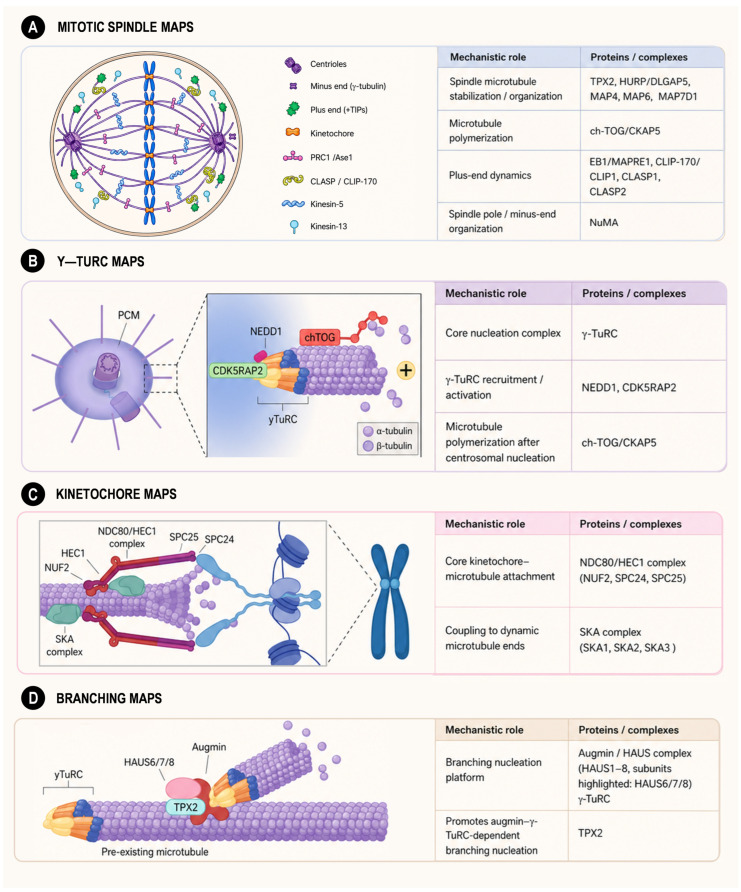
Mitotic microtubule-associated proteins (MAPs) and microtubule-binding assemblies in the spindle. Mitotic MAPs are shown according to their predominant site of action during spindle assembly and chromosome segregation. (**A**) Mitotic spindle MAPs: TPX2 promotes microtubule assembly around chromosomes, HURP/DLGAP5 stabilizes spindle microtubules and kinetochore fibers, the TACC3–ch-TOG–clathrin module reinforces kinetochore fibers, NuMA focuses and stabilizes spindle minus ends, MAP6 associates with subsets of spindle microtubules, MAP7D1 contributes to spindle microtubule stability, and Aurora A functions at centrosomes and spindle microtubules through TPX2-dependent recruitment and activation. Plus-end regulators including EB1, CLIP-170, and CLASP1/2 control the behavior of growing spindle microtubule plus ends and help couple microtubule dynamics to spindle architecture. PRC1 is shown on bridging-fiber overlaps, where it crosslinks antiparallel microtubules and contributes to spindle organization. (**B**) γ-TuRC MAPs: γ-TuRC nucleates and caps spindle microtubules, NEDD1 recruits γ-TuRC to centrosomes, CDK5RAP2 promotes γ-TuRC tethering and activation, and ch-TOG/CKAP5 promotes microtubule polymerization following centrosomal nucleation. (**C**) Kinetochore MAPs: the NDC80 complex forms the principal outer-kinetochore microtubule-binding interface, with the paired calponin homology (CH) domains of NDC80/HEC1 and NUF2 forming the main microtubule-binding head, whereas the SKA complex provides an additional load-bearing microtubule-binding layer that strengthens attachment to dynamic plus ends. (**D**) Branching MAPs: augmin targets γ-TuRC to pre-existing spindle microtubules to drive branching nucleation. Within augmin, HAUS6 provides a major anchoring interface with the mother microtubule, while HAUS7 and HAUS8 contribute to the formation and stabilization of the microtubule-binding module that positions γ-TuRC for daughter microtubule formation. TPX2 promotes augmin–γ-TuRC-dependent branching nucleation. Protein localization is schematic and indicates predominant mitotic sites of action rather than mutually exclusive localization.

While centrioles and basal bodies both act as stable microtubule-organizing structures, their function changes with cell-cycle stage and cellular context [78,80]. In many vertebrate cells in G1/G0, the mother centriole docks at the plasma membrane and becomes the basal body of a primary cilium, which typically has a (9 × 2 + 0) axonemal organization and primarily functions as a signaling organelle [78,80,132]. This should be distinguished from motile cilia, which are usually built around a (9 × 2 + 2) axoneme and occur in specialized ciliated cells where they drive fluid flow or cell motility [72,133]. When cells re-enter the cell cycle, the primary cilium is resorbed, allowing centrioles to resume their centrosomal role in mitotic spindle organization (Figure 6) [76,134]. In this context, microtubules transition from relatively stable, scaffolded arrays to highly dynamic polymers that must be nucleated, organized, and remodeled on short timescales [135,136]. This shift is accompanied by a distinct repertoire of MAPs, which, unlike many ciliary or centriolar MAPs, are optimized for dynamic regulation, rapid turnover, and force generation [132,137,138]. Among the best examples of mitotic MAP specialization is TPX2, a spindle assembly factor that promotes microtubule assembly around chromosomes [139,140,141,142]. TPX2 has multiple microtubule-binding elements and is now seen as more than a simple spindle microtubule binder, as it connects Ran/importin-dependent signaling to microtubule assembly around chromosomes and helps organize spindle architecture [140,141,142]. Recent work further suggests that different TPX2 microtubule-binding repeats support distinct spindle activities, reinforcing the idea that diversification within a single MAP can generate context-specific mitotic functions [142]. HURP (DLGAP5) is a MAP that contributes to spindle assembly by stabilizing microtubules and acts cooperatively with TPX2 [143,144,145,146]. Rather than functioning as a generic stabilizer throughout the cell, HURP is enriched on spindle microtubules, particularly kinetochore fibers, and helps reinforce microtubules as they are incorporated into the spindle [143,144,145].

Recent work in Xenopus egg extract further showed that HURP is required for RanGTP-induced branching microtubule nucleation, where TPX2 helps concentrate branching machinery and HURP stabilizes the resulting daughter microtubules [146] (Figure 6).

Spindle assembly also depends on factors that keep newly formed microtubules organized into stable bundles. A well-studied example is the Transforming Acidic Coiled-Coil protein 3 calponin homology domain-Tumor Overexpressed Gene-clathring (TACC3–ch-TOG–clathrin) complex, which has been shown to stabilize kinetochore fibers and thereby contribute to spindle robustness [147,148]. ch-TOG/CKAP5 belongs to the XMAP215 family of microtubule polymerases, whereas TACC3 and clathrin help form inter-microtubule bridges that organize and reinforce k-fiber bundles [147,148,149]. This complex illustrates a recurring theme in mitotic MAP biology: spindle architecture is often generated by multiprotein assemblies in which different components contribute polymerization, bundling, and mechanical stabilization [147,148,149]. Another critical spindle-building MAP is NuMA, which is essential for focusing microtubule minus ends and maintaining the structural integrity of the spindle poles [150,151,152,153]. NuMA is often discussed together with dynein, but its role is not limited to serving as a passive adaptor for motor recruitment. NuMA accumulates at spindle poles, helps organize minus ends, and recent work suggests that NuMA can mechanically reinforce the spindle independently of dynein binding [151,152,153]. Together, these studies highlight an intricate network of MAPs working together to drive spindle assembly by promoting microtubule formation, stabilizing selected microtubule subsets, and organizing those polymers into a bipolar array [142,146,148,153] (Figure 6).

MAP6 is an atypical MAP whose role in mitosis appears to stem from its ability to stabilize subsets of spindle microtubules. Early work showed that MAP6 associates specifically with mitotic spindle microtubules and with midbody microtubules in cultured cells, suggesting that MAP6 plays a role in supporting microtubules in mitosis [154]. Later research revealed the mechanistic background for this observation, and it was shown that MAP6 can interact with tubulin through its Mn modules and that MAP6 might stabilize microtubules by bridging adjacent tubulin heterodimers [155]. Trichoplein (TCHP) is another protein clearly linked to centrosome-dependent control of microtubules in dividing cells. TCHP localizes to centrioles and binds Odf2 and ninein and is required for efficient microtubule anchoring at the centrosome in proliferating cells [156]. More recent work shows that depletion of TCHP causes chromosome mis-segregation, DNA damage and chromosomal instability together with reduced Mad2 and Cyclin B1 levels, indicating that TCHP is important to ensure orderly progression from the spindle checkpoint into mitosis [157]. Intriguingly, recent comparative analyses have also proposed TCHP as a candidate MIP, raising the possibility that its role in centrosomal and ciliary microtubule organization extends beyond outer-surface anchoring [9]. Aurora A is the best-established mitotic regulator among mitotic MAPs. Aurora A accumulates at centrosomes and spindle microtubules and is required for centrosome maturation, mitotic entry, bipolar spindle assembly, and proper chromosome segregation [158,159]. A key part of Aurora A’s function in the mitotic spindle is due to its regulation by TPX2, which recruits Aurora A to spindle microtubules and activates the kinase in the spindle apparatus [160,161]. Interestingly, it was found that TCHP can directly activate Aurora A in proliferating cells to suppress primary cilium formation; however, this interaction is best established in centriolar ciliogenesis context rather than as a canonical spindle mechanism [162].

#### 4.3.1. γ-Tubulin Ring Complex (γ-TuRC) and Spindle Microtubule Nucleation

Another crucial spindle-associated microtubule regulator is the γ-tubulin ring complex (γ-TuRC), which differs from most MAPs because it does not primarily stabilize or remodel an existing polymer, but instead serves as a template for the formation of new ones [163,164]. γ-TuRC is a multi-subunit complex composed of γ-tubulin together with the γ-tubulin complex proteins (GCP2–GCP6) and associated factors including MZT1/2, which together assemble into a spiral template for microtubule nucleation. Its localization and activity are controlled by a family of conserved γ-TuRC receptors and anchoring proteins. In vertebrate centrosomes, proteins such as AKAP9, CEP192, Pericentrin and CDK5RAP2 cooperate to recruit, anchor and activate γ-TuRC, thereby regulating the spatial organization of microtubule nucleation [165]. Early work established that γ-TuRC nucleates microtubules from the periphery of centrosomes and can cap their minus ends [163,164,166]. γ-TuRC therefore provides the foundation for centrosomal microtubule organization. More broadly, these functions illustrate that microtubule-binding strategies in mitosis are not limited to lattice recognition or plus-end tracking but also include structural templates that define where a polymer can begin [167,168].

The mitotic spindle relies on regulated γ-TuRC recruitment rather than constitutive nucleation [169,170]. In vertebrate cells, NEDD1 is required to recruit γ-TuRC to centrosomes, while centrosomal proteins such as CDK5RAP2 help tether and activate the complex, rapidly increasing the nucleation capacity upon mitotic entry [169,170,171]. This is evolutionarily interesting because Centrosomin motif 1 (CM1)-containing γ-tubulin-complex receptors are conserved from fungi to mammals [172]. In mammals, CDK5RAP2 uses its γ-tubulin complex nucleation activator motif (γTuNA) motif to recruit and activate γ-TuRC [170,173], whereas related CM1 proteins in fungi promote assembly and activation of nucleation-competent γ-tubulin templates [174]. Together, these observations suggest that microtubule nucleation has diversified not only through changes in the core γ-tubulin complex, but also through accessory factors that specify its localization and activity [170,171] (Figure 6).

A second major feature of mitotic γ-TuRC specialization is its ability to be redeployed away from centrosomes. The augmin complex recruits γ-TuRC to pre-existing spindle microtubules, enabling microtubule-dependent microtubule nucleation within the body of the spindle [175,176,177]. In Xenopus egg extracts, this branching pathway is stimulated by TPX2 and generates daughter microtubules from the sides of mother microtubules, providing a mechanism to amplify spindle mass where it is most needed for chromosome segregation [176,177]. Recent work has further changed how γ-TuRC is viewed. Cryo-EM studies revealed that native human γ-TuRC is an asymmetric, multi-subunit assembly rather than a constitutively perfect 13-protofilament template. This finding helps explain why nucleation must be actively regulated [178,179]. More recent analyses of γ-TuRC-capped microtubules and CDK5RAP2-bound complexes further suggest that activation involves conformational changes that bring the γ-tubulin array into closer vicinity with the microtubule lattice [180,181].

Recent structural studies have provided important mechanistic insight into how augmin engages the mother microtubule [182,183]. Rather than associating with spindle microtubules through a diffuse electrostatic interface alone, augmin appears to use a composite microtubule-binding module within its tubulin-interacting interface/N-terminal clamp (TII/N-clamp) subcomplex [182,183,184]. Structural models predicted that HAUS6 and HAUS7 contain divergent calponin homology domains related to the NDC80/NUF2-like calponin homology (NN-CH) family, and that the conserved basic surface of HAUS6 overlaps with the tubulin-binding face found in NDC80-family microtubule binders [182]. Experimental testing subsequently showed that the conserved CH domain of HAUS6 is a major anchoring element for augmin, binding the inter-protofilament groove between adjacent β-tubulin subunits and orienting augmin on the mother lattice [183]. Together with the disordered N-terminus of HAUS8, this HAUS6-based attachment helps explain how augmin can stably dock on pre-existing spindle microtubules while positioning γ-TuRC to generate daughter microtubules at defined branch angles. Although centrosomes are the principal microtubule-organizing centers during mitosis, γ-TuRC also functions at non-centrosomal sites. In differentiated cells, microtubule nucleation occurs from organelles including the Golgi apparatus, where distinct γ-TuRC recruitment mechanisms generate polarized microtubule arrays. In these non-centrosomal networks, minus-end-binding proteins of the CAMSAP family stabilize free microtubule minus ends independently of centrosomes, thereby maintaining long-lived microtubule arrays. Small GTPase signaling pathways, including Arf GTPase regulators, further modulate γ-TuRC-dependent nucleation and organization at these alternative microtubule-organizing centers [185].

More recently, the functional repertoire of γ-TuRC has expanded beyond canonical centrosomal nucleation. In addition to its well-established role in augmin-dependent branching within the spindle, γ-TuRC also exists in a distinct augmin-associated pool within the centriole lumen [186]. This luminal augmin–γ-TuRC population does not primarily act as a cytoplasmic microtubule nucleator, but instead contributes to centriole integrity and maintenance of ciliogenesis competence, emphasizing that γ-TuRC can also function as a structural or regulatory assembly rather than only a nucleation template [186]. A newer study further suggests that centriole-lumenal augmin–γ-TuRC is protected during interphase and can be released in mitosis to aid chromosome alignment, linking this non-canonical pool back to spindle function [187].

#### 4.3.2. MAPs and Plus-End Microtubule Dynamics

If spindle assembly factors determine where and how spindle microtubules are built, plus-end regulators help determine how those microtubules behave once assembled [65,188,189,190,191]. This is especially important in mitosis, where microtubule plus ends must first explore the spindle space, then become captured by kinetochores, and later maintain load-bearing attachments while continuing to grow and shrink [192,193,194,195,196]. Proteins such as EB1, CLIP-170, and the CLASPs therefore occupy a central position in mitotic microtubule control [65,189,190,197,198,199,200,201] (Figure 6). Rather than being confined to one function, these proteins help create a dynamic interface between the growing microtubule end and its local environment, influencing rescue, persistence, and the recruitment of additional regulators [65,189,190,191,200,202,203]. Among these plus-end-associated factors, the CLASP proteins are especially informative because they act in multiple mitotic contexts, including the spindle, kinetochores, and the central spindle [199,201,202,203,204,205,206,207]. Their domain organization and broad conservation suggest that they have been repeatedly adapted to support both dynamic regulation and structural organization of microtubule arrays [191,206,207]. In the mitotic spindle, this flexibility is likely one reason why CLASPs bridge several phases of mitosis, contributing first to spindle dynamics and later to anaphase and cytokinetic microtubule organization [199,202,203,204,205,207]. The next major layer of specialization appears at the kinetochore–microtubule interface, where MAPs must convert highly dynamic polymers into load-bearing chromosome attachments without eliminating the capacity for error correction [199,200,201,203,205,206]. That interface is especially useful for an evolutionary discussion because it brings together classical microtubule binders, plus-end regulators, and large macromolecular attachment complexes into one highly specialized functional module [199,200,201,203,205,206].

Andersen et al. described a modular structural logic for ciliary microtubule inner proteins, and a similar principle appears to apply to plus-end regulators, which are built from a limited set of conserved interaction modules rather than a single shared “plus-end fold” [9]. One major example is the calponin homology domain of the EB proteins, which forms the core microtubule-binding module of EB1 and allows EB proteins to recognize the structural state of growing microtubule ends [208,209]. A second conserved module is the CAP-Gly domain found in proteins such as CLIP-170, whose plus-end tracking in mammalian cells depends on composite binding sites generated by EB1 and tyrosinated α-tubulin rather than on autonomous recognition of the growing end [65]. A third recurring theme is the TOG and TOG-like fold, which is used by XMAP215/ch-TOG family polymerases to bind soluble tubulin and promote polymerization, and by CLASP proteins to suppress catastrophe and promote rescue [210,211]. In addition, many plus-end regulators are recruited through short serine–any amino acid–isoleucine–proline (SxIP) motifs that bind the EB C-terminal homology domain, allowing them to hitchhike on the EB scaffold at growing ends [212]. Together, these examples show that the diversity of plus-end regulation arises from repeated use of a small number of conserved structural strategies that have been adapted for end recognition, tubulin handling, and EB-dependent recruitment.

#### 4.3.3. Kinetochore Microtubule Binding MAPs

The kinetochore-microtubule interface represents one of the most specialized examples of mitotic microtubule regulation. At this site, MAPs must convert highly dynamic spindle microtubules into attachments that are strong enough to bear force, yet still sufficiently labile to permit error correction. This creates a distinct regulatory problem compared with other microtubule arrays: kinetochore-associated MAPs cannot simply maximize stability, because stable but incorrect attachments are just as dangerous as unstable ones. Instead, the kinetochore uses a layered ensemble of microtubule-binding factors that together support initial capture, conversion to end-on attachment, persistence under load, and coupling to microtubule polymerization and depolymerization [192,213,214,215].

At the core of this interface is the NDC80 complex, the principal outer-kinetochore microtubule-binding assembly. Importantly, the microtubule-binding head is formed not by NDC80/HEC1 alone, but by the paired calponin homology domains of NDC80 and its related partner NUF2. Early biochemical and structural work showed that the Ndc80–Nuf2 heterodimer binds microtubules directly and that these tightly associated calponin homology domains create the major microtubule-binding surface of the complex [213,216,217]. Functional dissection further showed that the calponin homology domains of both Hec1/NDC80 and Nuf2 make distinct contributions to stable kinetochore–microtubule attachment, indicating that NUF2 is not merely a structural support subunit but an active part of the microtubule-binding interface [217]. Classical biophysical studies later showed that multiple NDC80 complexes can work together to stay attached to dynamic microtubule tips while still bearing load, through a mechanism of biased diffusion [192]. Subsequent cryo-EM studies showed that NDC80 complexes can assemble into cooperative arrays along the microtubule lattice, providing a structural explanation for how these attachments remain robust under tension [218] (Figure 6).

However, NDC80 alone does not explain the full behavior of mature kinetochore attachments. The SKA complex provides an additional microtubule-binding layer that cooperates with NDC80 to strengthen and stabilize kinetochore–microtubule interactions, particularly at dynamic plus ends [219,220,221,222,223] (Figure 6). Thus, the mature outer kinetochore is best viewed as a cooperative, multivalent attachment system rather than a single microtubule linker. Moreover, the behavior of kinetochore fibers is influenced not only by factors at the kinetochore itself, but also by the surrounding spindle architecture. PRC1 provides a useful example. Best known for organizing and bundling antiparallel microtubule overlaps [224], PRC1 crosslinks microtubules within bridging fibers in metaphase spindles, thereby helping mechanically couple sister kinetochore fibers [225]. PRC1-crosslinked overlap bundles also emerge during spindle assembly and are reorganized into more distinct bundles near kinetochores and chromosomes [226]. Acute PRC1 removal partially disassembles bridging fibers and impairs chromosome alignment, indicating that accurate congression depends not only on end-on coupling at kinetochores, but also on force transmission through PRC1-organized overlap bundles within the spindle body [227] (Figure 6).

A more recently implicated factor is MAP7D1. Although MAP7D1 is not a canonical outer-kinetochore component, it is emerging as a mitotic microtubule regulator that contributes to spindle robustness. Earlier work showed that MAP7D1 helps maintain acetylated stable microtubules [228], and a 2023 study further linked MAP7 and MAP7D1 to cell-cycle control by showing that they promote DNA double-strand break repair in G1 and support G1 progression [229]. Consistent with a more direct role in mitosis, a 2025 study found that a MAP7D1 loss-of-function mutation disrupts microtubule association, reduces microtubule density, and causes unstable bipolar or multipolar spindles, lagging chromosomes, and shortened inter-centrosomal distance [230]. Together, these observations suggest that kinetochore–microtubule regulation is shaped not only by canonical outer-kinetochore binders such as NDC80, NUF2, and SKA, but also by spindle-architectural proteins such as PRC1 and broader microtubule-stabilizing factors such as MAP7D1 that help define the mechanical environment in which chromosome segregation occurs.

## 5. MAPs in Neurons

**Figure 7:**
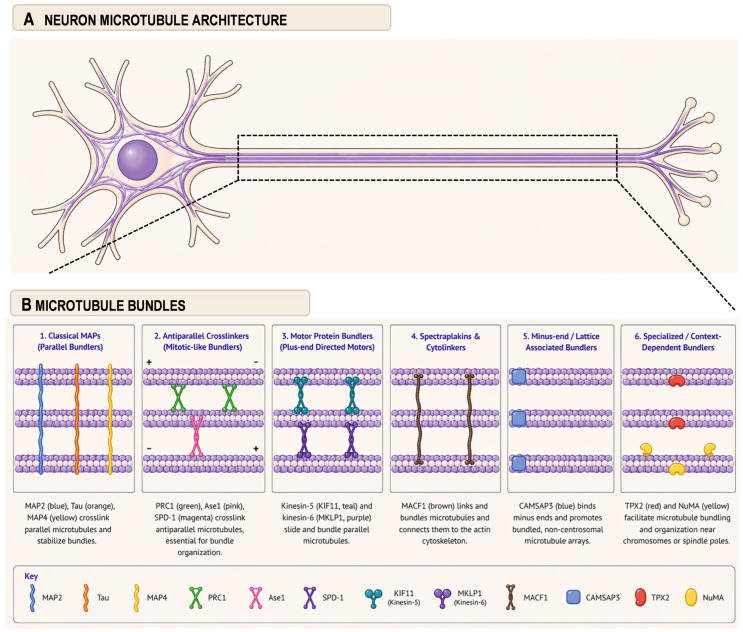
Neuronal microtubule organization and bundling mechanisms. (**A**) Neuron ultrastructure. Schematic of a neuron highlighting the soma, dendrites, and axon. Microtubules form long, polarized bundles that extend along the axon and support intracellular transport and structural integrity. (**B**) Microtubule bundlers. Overview of major classes of proteins that organize and stabilize microtubule bundles. (1) Classical MAPs (e.g., MAP2, Tau, MAP4) crosslink parallel microtubules and stabilize bundles. (2) Antiparallel crosslinkers (e.g., PRC1, Ase1, SPD-1) organize antiparallel arrays. (3) Motor protein bundlers (e.g., kinesin-5/KIF11 and kinesin-6/MKLP1) slide and bundle microtubules. (4) Spectraplakins and cytolinkers (e.g., MACF1) connect microtubules to the actin cytoskeleton. (5) Minus-end/lattice-associated proteins (e.g., CAMSAP3) stabilize non-centrosomal microtubule arrays. (6) Specialized/context-dependent bundlers (e.g., TPX2, NuMA) contribute to microtubule organization in specific cellular contexts such as mitosis.

Compared with cilia and centrioles, neuronal microtubules are decorated by a broader and more dynamic MAP repertoire. A useful organizing principle is that neuronal MAPs fall into four overlapping functional classes: classical lattice binders that control spacing and dynamics, stabilizers of long-lived axonal bundles, neuron-enriched assembly factors that promote tubulin incorporation, and tip or branch-associated MAPs that help microtubules invade growth cones and nascent branches. Within that framework, the core neuronal MAP set includes tau/MAPT, MAP1A, MAP1B, MAP2, MAP4 in some neuronal contexts, MAP6/STOP, the MAP7 family including MAP7, MAP7D1, MAP7D2 and MAP7D3, MAP8, the CRMP1-5 family, and recently the luminal factors JPT1/2 [12,15,231,232,233,234,235,236,237,238] (Figure 7). Additional direct neuronal microtubule binders that are worth discussing, even if they are sometimes treated separately from the classical MAP canon, include doublecortin (DCX) and related DCLK proteins, as well as branch- and plus-end-associated binders such as CLASP1/2 and APC (see above). The classical neuronal MAPs still provide the conceptual backbone for this field. Tau and MAP2 are the canonical members of the MAP2/tau family, using short P-G-G-G motif repeat regions to bind along the outer microtubule lattice and projecting N-terminal regions outward [239,240,241,242], thereby influencing spacing, mechanics, and interactions with motors and actin-associated systems.

Recent evidence indicates that tau should no longer be described simply as a generic stabilizer of axonal microtubules [243]. Work in neurons showed that tau is enriched on the labile domain of axonal microtubules and promotes assembly while limiting access of stronger stabilizers such as MAP6, rather than acting as the principal long-term stabilizer itself [235,244,245]. That makes tau functionally distinct from older textbook descriptions and places it closer to a modulator of dynamic axonal microtubule behavior. MAP2 is enriched in dendrites, whereas tau is strongly associated with axons [246,247], although this partition is not absolute. MAP1A and MAP1B are larger, multidomain MAPs that associate with microtubules and also couple to actin-related systems through their light chains [248,249,250,251], making them especially relevant to neurite extension and growth cone behavior. MAP8 (MAP1S) is less often foregrounded in neuron reviews, but it is a bona fide MAP with two mapped microtubule-binding regions and should be included when aiming for completeness [252]. MAP8 shows a highly dynamic localization pattern, associating with microtubules and mitotic spindles while remaining diffusely cytoplasmic during interphase. Notably, it preferentially associates with stabilized microtubules and colocalizes with RASSF1A. Upon apoptotic signaling, MAP1S relocalizes to perinuclear punctate structures corresponding to mitochondrial aggregates and is also detected in the nucleus, highlighting a potential link between cytoskeletal regulation and stress-responsive pathways [253]. MAP4 is not neuron-specific in the way tau or MAP2 is, but it is a direct lattice binder [233,254,255].

MAP6 stands apart from tau because it behaves as a more authentic stabilizer of long-lived neuronal microtubules [256]. MAP6 is best known for conferring cold and drug resistance and for supporting stable axonal microtubule populations. More recent reviews emphasize that MAP6 also broadens the definition of a neuronal MAP because its effects extend beyond simple lattice coating, influencing microtubule architecture, neuronal connectivity, and synaptic function. The MAP7 family deserves a full section because it has emerged as one of the clearest links between direct lattice binding, kinesin regulation, and axon branching. MAP7 binds microtubules through an N-terminal MBD and also recruits or activates kinesin-1 through its C-terminal region. Structural work published in 2024 refined how MAP7 associates with the lattice and how its MBD behaves dynamically on microtubules [257]. Functionally, MAP7 promotes axon collateral branch development and helps prevent retraction of nascent branches by stabilizing microtubules in those branches. Thus, MAP7 is not simply a passive lattice binder, but a branch-maturation MAP that also influences transport within developing axonal branches [258]. The MAP7 paralogs extend this idea of spatial specialization. MAP7D2 localizes strongly to the proximal axon, where it promotes kinesin-1 entry and cargo trafficking, while MAP7D1 and MAP7D2 can both contribute to microtubule stabilization by distinct mechanisms [258,259]. This makes the MAP7 family especially relevant when discussing how neurons regionalize their microtubule surface for different transport and morphogenetic outcomes.

Previous studies have highlighted the role of Collapsin-Response-Mediator Proteins (CRMPs) in neuronal dendritic and axonal compartments [260]. CRMP2 is the best characterized member of this family and acts as an important link between extracellular guidance signals and the microtubule cytoskeleton. It promotes microtubule assembly by binding tubulin heterodimers and supports axon formation, neurite extension, and growth cone dynamics [237,260]. Unlike classical lattice-binding MAPs, CRMP2 functions mainly as a regulated tubulin-binding assembly factor, with its activity controlled by phosphorylation downstream of guidance cues. CRMP4 has also been implicated in cytoskeletal remodeling during neurite outgrowth and growth cone responses, often functionally intersecting with CRMP2, although its direct microtubule-regulatory mechanism is less well resolved. Thus, CRMP2 can be considered the prototypic microtubule assembly-promoting CRMP, while CRMP4 and other family members contribute more broadly to neuronal morphogenesis and cytoskeletal regulation [260].

Recent work has also expanded the neuronal MAP landscape to include luminal microtubule-associated proteins. JPT2 was recently identified as a taxane-sensitive microtubule-lumen protein that modulates the accessibility of MEC17/αTAT1 to the microtubule lumen [261]. JPT1 appears to share related sequence features with JPT2, including tau-like C-terminal repeat motifs containing a conserved PPGGK/S sequence within flexible regions. These motifs are predicted to engage a luminal microtubule-binding pocket, consistent with the reported competition between JPT2 and paclitaxel [261]. However, the direct luminal microtubule-binding role of JPT1 remains less well established than that of JPT2 and should therefore be interpreted more cautiously.

### MAPs in Axonal Branching and Growth Cones

A major conceptual advance in neuronal cytoskeleton biology has been the recognition that microtubules can be generated de novo from pre-existing microtubules, rather than exclusively from centrosomal templates. This process, termed microtubule branching nucleation, is now established as a key contributor to axonal arborization and growth cone remodeling. In metazoa, branching relies on a conserved core machinery centered on the augmin complex and the gamma-tubulin ring complex (γ-TuRC) [177,262,263,264,265,266] but is increasingly understood to involve additional MAPs that directly engage and modify the microtubule lattice (see below). The augmin complex acts as a bridge between γ-TuRC and the template MT, thereby recruiting the nucleation unit to its correct location. At the heart of this system lies the augmin–γ-TuRC module, which enables templated nucleation from existing microtubules. Augmin binds laterally to a “mother” microtubule through direct lattice contacts and recruits γ-TuRC to this site. In doing so, it positions γ-TuRC such that a new “daughter” microtubule is nucleated at a defined angle relative to the original filament. The augmin attachment structure on microtubules resembles that of NDC80 bound to the MT, with the conserved CH domain of augmin’s HAUS6 subunit directly proximal to the MT lattice [262]. This mechanism allows neurons to locally amplify their microtubule network within axons and dendrites, independently of centrosomes. Importantly, augmin does more than recruit a nucleator. By defining the spatial orientation of γ-TuRC, it effectively converts a segment of the microtubule lattice into a nucleation-competent platform, functioning analogously to a spatially restricted MAP that encodes geometry onto the polymer (Figure 6).

In parallel with this templated mechanism, a second mode of branching has emerged based on direct lattice remodeling. The protein SSNA1 (NA14) exemplifies this pathway. SSNA1 binds directly to centriole and cytoplasmic microtubules and assembles into coiled-coil structures along the lattice [267], where it appears to induce structural changes that promote the formation of new microtubule ends and regulate cell division [268]. Mechanistically, in the context of microtubule branching, SSNA1 has been proposed to stabilize protofilament curvature or lattice defects, thereby facilitating microtubule splitting and the emergence of branch-like structures [269]. In this context, SSNA1 may act either upstream of or in parallel with augmin-dependent nucleation, introducing a conceptually distinct paradigm in which branching arises from lattice destabilization and reorganization, rather than templated nucleation alone. Once nascent microtubules are generated, their persistence and functional integration depend on a second layer of MAPs that stabilize and guide branch growth. Members of the MAP7 family and DCX/DCLK proteins stabilize nascent branches by binding the microtubule lattice, reducing catastrophe, and promoting persistence of newly formed microtubules [270,271].

Taken together, these findings support an emerging model in which neuronal microtubule branching arises from two partially overlapping mechanistic classes. In the first, templated nucleation via the augmin–γ-TuRC pathway generates new microtubules from existing lattices. In the second, lattice remodeling driven by SSNA1 and related factors produces new microtubule ends through structural reorganization.

## 6. Diseases Associated with MAPs Across Systems

The expanding catalog of MAPs has revealed that defects in these proteins do not simply destabilize microtubules but disrupt highly specific architectural and regulatory modules. Across neurons, cilia, and centrioles, disease phenotypes increasingly reflect failures in microtubule patterning, branching, and spatial organization, rather than generic loss of polymer integrity. Across systems, a unifying theme emerges. Diseases associated with MAPs are rarely caused by simple loss of microtubules. Instead, they reflect disruption of specific microtubule architectures and spatial organization [272,273]. In neurons, defects affect dynamic regulation, branching, and transport [274,275]. In cilia, defects disrupt axonemal patterning and motility, and in centrioles, defects impair structural integrity, centrosome assembly, and chromosome segregation [276,277].

As the catalog of MAPs continues to expand, particularly with the discovery of lumenal proteins and branching factors, additional disease links will likely emerge, further emphasizing that microtubules are not passive structures but highly organized platforms whose integrity depends on complex MAP networks.

### 6.1. Ciliopathies and Axonemal MAP Defects

Ciliary MAPs are strongly associated with a range of ciliopathies, reflecting the structural and regulatory complexity of axonemal microtubules. Defects in axonemal MAP systems are associated with a range of disorders, including primary ciliary dyskinesia, Joubert syndrome (e.g., CSPP1, CEP104, TOGARAM1, and KIF7), retinitis pigmentosa (e.g., FAM161A), and situs inversus (for comprehensive reviews see [133,278,279]), among others [280,281]. While many causative genes encode dynein arms or assembly factors, increasing evidence implicates structural MAPs, including MIPs and outer-surface complexes, in maintaining proper axonemal function [10,282,283,284]. Disruption of these MAPs leads to defects in microtubule spacing, radial spoke interactions, and central pair regulation, ultimately impairing coordinated beating in motile (9 × 2 + 2) cilia. Defects in central pair architecture are a known cause of motile ciliopathies, indicating that specialized lattice-binding proteins at the seam and outer surface are critical for function [113,115].

### 6.2. The Elusive Link Between Centriole MAPs, Cell Division, and Cancer

Centrioles and centrosomes have long occupied a prominent place in cancer research because of their obvious importance for spindle assembly and cell division, and because abnormalities in centrosome number or structure are common in human tumors [285,286,287]. Yet their precise place in carcinogenesis remains unresolved. The key question is still whether centrosome defects act as genuine drivers of transformation or instead arise secondarily as tumors acquire broader cell-cycle and genomic instability, which leaves the field with a classic chicken-and-egg problem [288,289].

At present, the strongest evidence points to centrosome amplification, rather than centrosome loss, as the more plausible cancer-promoting lesion. Extra centrosomes can increase merotelic kinetochore-microtubule attachments and chromosome mis-segregation, even when cells ultimately cluster their centrosomes into a bipolar spindle, and experimentally induced centrosome amplification is sufficient to promote tumorigenesis in animal models [290,291,292]. By contrast, centrosome depletion has not emerged as an equally clear oncogenic driver. In mammalian cells, centrosome loss often activates a 53BP1-USP28-p53-dependent mitotic surveillance pathway rather than conferring a proliferative advantage [293]. This more limited impact is consistent with the classic Drosophila DSas-4 model, where acentriolar flies develop with nearly normal timing despite slower spindle assembly and some abnormal neuroblast divisions. Rather than causing a dramatic block in cell division, centriole loss primarily disrupts cilia and flagella formation, leading to death shortly after eclosion [294].

Defects in MAPs contribute to several human diseases. In the nervous system, the best-known example is tau, whose abnormal phosphorylation, aggregation, and loss of normal microtubule-regulating function are central features of tauopathies and other neurodegenerative disorders [295]. In cancer, MAP dysregulation is also highly relevant. Because microtubules are essential for mitosis, intracellular organization, trafficking, and cell migration, alterations in the proteins that control them can strongly influence tumor-cell proliferation, survival, and invasive behavior [296,297]. This is particularly important for mitotic MAPs, because disruption of spindle-assembly and chromosome-segregation pathways can promote chromosomal instability and thereby support tumor evolution, heterogeneity, and therapy resistance [297,298,299]. For example, dysregulation of TPX2, NuMA, HURP/DLGAP5, and KIF2C has been linked to spindle defects, chromosome mis-segregation, and chromosomal instability (CIN) [300,301,302,303]. CIN is itself a hallmark of cancer and is closely associated with tumor evolution, intratumoral heterogeneity, metastasis, and treatment resistance [304]. MAP dysregulation can also affect how cancer cells move and adapt to their environment. STMN1 has been associated with aggressive behavior, poor outcome, and, in some settings, chemoresistance, while altered EB1/MAPRE1 expression has been linked to poor prognosis in colorectal cancer and glioblastoma. CLIP-170 has also been implicated in migratory and invasive behavior in specific cancer models [305,306,307,308]. Since many anticancer drugs target microtubules, MAP dysregulation can additionally influence treatment response. High STMN1 expression has been associated with chemoresistance, the CLIP-170S variant with taxane resistance, and HURP with reduced sensitivity to vinca alkaloids [305,309,310,311]. At the same time, these dependencies suggest therapeutic opportunities, as tumors that rely on specific MAP-regulated programs may be selectively vulnerable to their disruption [297,309].

### 6.3. Neurodegenerative and Neurodevelopmental Disorders

Among all MAPs, tau (MAPT) is the most extensively linked to human disease [312]. Mutations and pathological aggregation of tau underlie a spectrum of tauopathies, including frontotemporal dementia and Alzheimer’s disease [313,314]. Mechanistically, tau pathology leads to loss of its normal microtubule-binding function combined with toxic gain-of-function aggregation. Disease phenotypes correlate not only with microtubule destabilization but also with impaired axonal transport and cytoskeletal organization [315,316,317], reinforcing the idea that tau regulates microtubule accessibility and dynamics rather than acting as a simple stabilizer.

The microtubule-binding protein DCX is directly linked to severe neurodevelopmental disorders [318,319]. Mutations in DCX cause lissencephaly and subcortical band heterotopia, reflecting its essential role in stabilizing microtubules during neuronal migration. These phenotypes highlight the importance of straight microtubule stabilization and growth cone dynamics, processes now understood to depend on DCX-mediated lattice binding. MAP6 (also known as STOP) not only stabilizes microtubules but also participates in synaptic function, vesicle dynamics, and receptor homeostasis. Disruption of MAP function can therefore have profound consequences, particularly in the brain, where precise cytoskeletal regulation underpins connectivity and plasticity. Indeed, alterations in MAP expression or function have been linked to a range of neurological and psychiatric disorders, including schizophrenia and neurodevelopmental conditions. Together, these findings position MAPs as central hubs that coordinate cytoskeletal dynamics with cellular signaling, and highlight their importance as both mechanistic drivers and potential therapeutic targets in human disease [320]. Similarly, the CRMP family is implicated in multiple neurological conditions, including neurodegeneration and psychiatric disorders. CRMP2 dysfunction affects microtubule assembly and axon guidance, linking disease phenotypes to impaired tubulin incorporation and cytoskeletal remodeling downstream of signaling pathways [320].

## 7. Conclusions

Taken together, these studies make it clear that MAPs are much more than helper proteins. Across centrioles, cilia, spindles, and neurons, they are central to how different microtubule systems are built, organized, and tuned for function. What is emerging is not just a list of isolated factors, but a broader logic in which similar binding strategies are reused in different contexts and adapted to distinct microtubule architectures. Understanding how these proteins work together, and what happens when they fail, will be important for explaining both cytoskeletal specialization and disease.

## Figures and Tables

**Table 1:** Classification of microtubule-associated proteins and related structural regulators discussed in this review. Proteins and protein complexes discussed throughout the review are grouped into broad functional categories according to their principal association with microtubule-based structures. Well-supported MAPs comprise proteins or complexes with established microtubule-binding, organizing, stabilizing, depolymerizing, motor, or regulatory functions. MIPs are defined here as specialized MAPs associated primarily with the microtubule lumen, inner wall, or inner-junction axonemal structures. MOPs and other outer-surface-associated proteins comprise proteins or complexes associated with the outer microtubule surface, the microtubule seam, central-pair projections, or outer axonemal/ciliary structures. The final category includes structural regulators that contribute to the assembly, positioning, recruitment, or regulation of microtubule-based structures but are not classified here as direct MAPs, MIPs, or MOPs. This classification is intended as a simplified framework for this review, and several proteins have context-dependent functions that could place them in more than one category.

Classification	Definition Used in This Table	Proteins/Complexes Mentioned in the Review (Alphabetical)
**Well-supported MAPs**	Proteins or complexes with direct or well-established microtubule-binding, -organizing, -stabilizing, -depolymerizing, motor, or regulatory functions, excluding proteins listed separately as MIPs or MOPs.	APC, Ase1/SPD-1, ASPM, augmin/HAUS complex, CAMSAP1, CAMSAP2, CAMSAP3, ch-TOG/CKAP5/XMAP215, CLASP1/2, CLIP-170, CRMP family, CRMP1, CRMP2, CRMP3, CRMP4, CRMP5, cytoplasmic dynein, DCX/DCLK proteins, DLGAP5/HURP, EB1/MAPRE1, EB1–3/MAPRE1–3, HAUS6, HEC1/NDC80, HOOK1–3, KIF11/kinesin-5, KIF13B, KIFC1/kinesin-14, kinesin-1, kinesins, MACF1, MAP1A, MAP1B, MAP2, MAP4, MAP6/STOP/SAXO, MAP7, MAP7D1, MAP7D2, MAP7D3, MAP8/MAP1S, MCAK, MKLP1/kinesin-6, NDC80 complex, NuMA, NUF2, p150^Glued, PRC1, SKA complex, spastin, SSNA1/NA14, TACC3, TACC3–ch-TOG–clathrin complex, Tau/MAPT, TPX2.
**MIPs/luminal MAPs**	Specialized MAPs located mainly within the microtubule lumen, along the inner wall, or in luminal/inner-junction axonemal structures.	C10orf82, C11ORF1/CFAP68, CCDC105, CFAP20/FAP20, CFAP52/FAP52, CFAP68/C11ORF1, CFAP77, CFAP95, CFAP106/FAP106, CFAP107, CFAP126/FAP126, CFAP161, CFAP276/FAP276, EFHB, FAM161A, FAM166 family, FAP45, FAP53, FAP127, JPT1, JPT2, PACRG–CFAP20 inner-junction module, RIB72A/B, RIBC1/2, SPACA9, SPAG8, TEKT5, TEKTIP1, TEKTL1, tektins/TEKT1–5, WDR90/POC16.
**MOPs/outer-surface or projection-associated MAPs**	Specialized MAPs or MAP complexes associated mainly with the outer microtubule surface, the microtubule seam, central-pair projections, or outer axonemal/ciliary structures.	axonemal dynein arms, CIMAP2, CIMAP3, CFAP97D1, DRC/dynein regulatory complex, EFCAB3, FAP47, FAP59/FAP172 molecular-ruler complex, Hydin, nexin–dynein links, ODA-docking complex, pixin, pixin regulatory complex, radial spoke complexes, SPEF1, SPMAP1, SPMAP2, TLP1, TLP2, TPPP, TPPP-like proteins.
**MAP-associated structural regulators**	Proteins or complexes that help build, position, recruit, or regulate microtubule-based structures, but are not classified here as direct MAPs, MIPs, or MOPs.	ARMC4, Aurora A, calaxin, CCDC15, CCDC39/CCDC40, CDK5RAP2, centrin/Centrin-2, CEP83, CEP120, CEP135/Bld10, CEP164, CEP295, clathrin, CP110, CPAP/CENPJ, FhaB, γ-tubulin, γ-TuRC, γ-TuSC, HYLS1, NEDD1, NME7, POC1A, POC1B, POC5, RTTN, SAS-6, SCLT1, SPICE1, STIL, TCHP/Trichoplein, TSSK-associated structures.
**Tubulin-code enzymes and related regulators**	Enzymes and regulatory proteins that write, erase, or interpret tubulin post-translational modifications and thereby influence MAP recruitment or microtubule identity.	ATAT1, CCP/AGBL family enzymes, HDAC6, MATCAP, SETD2, SIRT2, TTL, TTLL enzymes, VASH1/2–SVBP.

**Table 2:** Major microtubule-binding domains, motifs, and modules discussed in this review. The table summarizes selected MBDs, motifs, and structural modules referred to throughout the review. For each entry, representative proteins or complexes are listed together with the main binding mode, cellular context, and functional relevance. The table is intended as a guide to the recurring binding strategies used by MAPs, MIPs, MOPs, and related regulators across specialized microtubule systems, rather than as a complete catalog of all known MBDs. Some modules are defined by well-characterized structural domains, whereas others represent repeated motifs, short linear interaction motifs, or scaffold-based binding modes.

Microtubule-Binding Interface or Recognition Module	Main Binding Mode/Lattice Feature	Representative Proteins or Complexes
**ASH/MSP domain**	Structural domains positioned on or near the outer microtubule surface in central-pair projection complexes	ASPM, NPHP4, DLEC1, CEP192, Hydin, TRAPPII complex members, OCRL, SPAG17, CFAP221, related central-pair projection proteins
**Basic microtubule-binding region**	Intrinsically disordered Lys/Arg-rich regions bind electrostatically along the negatively charged outer microtubule lattice and promote stabilization and bundling	MAP1A, MAP1B, MAP4
**Calponin homology (CH) domain**	Binds the microtubule lattice or growing microtubule ends	EB1-3/MAPRE1-3, SPEF1, HOOK1-3
**CAP-Gly domain**	Recognizes the C-terminal EEY/F motif of tyrosinated α-tubulin and composite EB/tubulin-binding sites	CLIP-170, KIF13B, p150^Glued
**CKK domain**	Conserved C-terminal domain that recognizes microtubule minus ends and stabilizes non-centrosomal microtubule arrays	CAMSAP1, CAMSAP2, CAMSAP3
**CM1/γTuNA motif**	Recruits and activates γ-tubulin nucleation complexes rather than binding the lattice directly	CDK5RAP2 and related γ-TuRC receptors
**Coiled-coil scaffold modules**	Extended coiled-coil regions form structural supports, crosslinks, or lattice-associated assemblies	NuMA, SSNA1/NA14, TACC3–ch-TOG–clathrin complex, MNS1, CFAP141, CFAP53, Tektins
**DM10**	Conserved luminal microtubule-binding domain that recognizes the inner α/β-tubulin lattice at the interdimer interface and contributes to microtubule stabilization.	RIB72A, RIB72B, CAPS2
**Doublecortin (DC) domain**	Recognizes the microtubule lattice at the interface between adjacent protofilaments and stabilizes polymerized microtubules	DCX, DCLK1, DCLK2
**GFG repeats**	Glycine–phenylalanine–glycine repeat modules bind the luminal lattice, often near seam-associated regions	CFAP77, EFHB
**Luminal scaffold/inner-wall binding module**	Bind or localize to the microtubule wall or lumen; precise domain architecture varies by protein	CCDC105, FAM161A, JPT2, SPACA9, WDR90/POC16
**MAP7 microtubule-binding domain**	N-terminal microtubule-binding region associates with the outer microtubule lattice	MAP7, MAP7D1, MAP7D2, MAP7D3
**Mn repeat module**	Repeated luminal tubulin-binding units contact tubulin heterodimers from inside the microtubule	MAP6/SAXO-family proteins and related ciliary MIPs
**Motor ATPase domain**	ATP-dependent motor domain that alternates between strong and weak affinity states to generate directional movement along microtubules	Cytoplasmic dynein, kinesins
**NN-CH-like domain**	CH-like fold adapted for lattice binding, including inter-protofilament grooves	HAUS6/augmin complex, HAUS6, NDC80/HEC1, NUF2
**NWE seam-binding module**	Specialized module recognizing heterotypic lattice contacts at the A-tubule seam	CFAP68/C11ORF1, CFAP95, CFAP107, CFAP161
**PYG repeats**	Short repeat modules contacting adjacent tubulin subunits from the microtubule lumen	C10orf82, FAM166 family
**SxIP motif**	Short linear motif binding the EB C-terminal domain tmediate plus-end tracking	EB-binding + TIPs
**Tau/MAP2-family repeats**	Conserved repeat regions bind longitudinally along the outer microtubule lattice	MAP2, MAP4, Tau/MAPT, JPT1/2
**TOG/TOG-like domain**	Bind curved or soluble tubulin dimers and regulate microtubule polymerization dynamics	CLASP1/2, XMAP215/ch-TOG/CKAP5
**TPPP-like domain**	Bind the outer surface of central-pair microtubules and may recognize curved or non-canonical lattice geometry	Other TPPP-like proteins, TLP1, TLP2
**Tubulin C-terminal tail recognition module**	Recognize the flexible α- or β-tubulin C-terminal tails, often in a post-translational modification-dependent manner	CAP-Gly proteins, HYLS1, spastin, TTLL/TTL-related enzymes

## Data Availability

No new data were created or analyzed in this study.

## Abbreviations

**Abbreviation**	**Meaning**
ASH	ASPM-SPD-2-Hydin domain
CAP-Gly	Cytoskeleton-associated protein glycine-rich domain
CH	Calponin homology domain
CIMAP	Ciliary microtubule-associated protein
CIN	Chromosomal instability
CM1	Centrosomin motif 1
CP	Central pair
DMT/DMTs	Doublet microtubule(s)
DNA	Deoxyribonucleic acid
DRC	Dynein regulatory complex
EEY/F	C-terminal glutamate–glutamate–tyrosine/phenylalanine motif
FtsZ	Filamenting temperature-sensitive mutant Z
GFG	Glycine–phenylalanine–glycine repeat/module
γ-TuRC	γ-tubulin ring complex
γTuNA	γ-tubulin complex nucleation activator motif
LECA	Last eukaryotic common ancestor
MAP/MAPs	Microtubule-associated protein(s)
MBD	Microtubule-binding domain
MIP/MIPs	Microtubule inner protein(s)
Mn	MAP6/SAXO-type Mn module repeat
MOP/MOPs	Microtubule outer protein(s)
MSP	Major sperm protein domain family
MT	Microtubule
NN-CH	NDC80/NUF2-like calponin homology domain
NWE	NWE seam-binding module/motif
O-GlcNAc	O-linked N-acetylglucosamine
PTM	Post-translational modification(s)
PYG	Proline–tyrosine–glycine repeat
SxIP	Serine–any amino acid–isoleucine–proline EB-binding motif
TII/N-clamp	Tubulin-interacting interface/N-terminal clamp subcomplex
TACC3	Transforming Acidic Coiled-Coil protein 3
TOG	Tumor overexpressed gene domain
TTL-like	Tubulin tyrosine ligase-like

## References

[1] Knossow M., Campanacci V., Khodja L.A., Gigant B. (2020). The Mechanism of Tubulin Assembly into Microtubules: Insights from Structural Studies. iScience.

[2] Alfaro-Aco R., Petry S. (2015). Building the Microtubule Cytoskeleton Piece by Piece. J. Biol. Chem..

[3] Barlan K., Gelfand V.I. (2017). Microtubule-Based Transport and the Distribution, Tethering, and Organization of Organelles. Cold Spring Harb. Perspect. Biol..

[4] McIntosh J.R., Grishchuk E.L., West R.R. (2002). Chromosome-microtubule interactions during mitosis. Annu. Rev. Cell Dev. Biol..

[5] Etienne-Manneville S. (2013). Microtubules in cell migration. Annu. Rev. Cell Dev. Biol..

[6] Blacque O.E., Scheidel N., Kuhns S. (2018). Rab GTPases in cilium formation and function. Small GTPases.

[7] Goodson H.V., Jonasson E.M. (2018). Microtubules and Microtubule-Associated Proteins. Cold Spring Harb. Perspect. Biol..

[8] Akhmanova A., Steinmetz M.O. (2015). Control of microtubule organization and dynamics: Two ends in the limelight. Nat. Rev. Mol. Cell Biol..

[9] Andersen J.S., Vijayakumaran A., Godbehere C., Lorentzen E., Mennella V., Schou K.B. (2024). Uncovering structural themes across cilia microtubule inner proteins with implications for human cilia function. Nat. Commun..

[10] Gui M., Orbach R. (2025). Microtubule inner proteins—Bridging structure and function in ciliary biology. J. Cell Sci..

[11] Legal T., Joachimiak E., Parra M., Peng W., Tam A., Black C., Guha M., Nguyen C.A., Ghanaeian A., Valente-Paterno M. (2025). Structure of the ciliary tip central pair reveals the unique role of the microtubule-seam binding protein SPEF1. Curr. Biol..

[12] Weingarten M.D., Lockwood A.H., Hwo S.Y., Kirschner M.W. (1975). A protein factor essential for microtubule assembly. Proc. Natl. Acad. Sci. USA.

[13] Janke C., Magiera M.M. (2020). The tubulin code and its role in controlling microtubule properties and functions. Nat. Rev. Mol. Cell Biol..

[14] Roll-Mecak A. (2020). The Tubulin Code in Microtubule Dynamics and Information Encoding. Dev. Cell.

[15] Sloboda R.D., Dentler W.L., Rosenbaum J.L. (1976). Microtubule-associated proteins and the stimulation of tubulin assembly in vitro. Biochemistry.

[16] Slep K.C. (2010). Structural and mechanistic insights into microtubule end-binding proteins. Curr. Opin. Cell Biol..

[17] Erickson H.P. (2007). Evolution of the cytoskeleton. Bioessays.

[18] Lowe J., Amos L.A. (1998). Crystal structure of the bacterial cell-division protein FtsZ. Nature.

[19] Wickstead B., Gull K. (2011). The evolution of the cytoskeleton. J. Cell Biol..

[20] Dehmelt L., Halpain S. (2005). The MAP2/Tau family of microtubule-associated proteins. Genome Biol..

[21] Kalcheva N., Albala J., O’Guin K., Rubino H., Garner C., Shafit-Zagardo B. (1995). Genomic structure of human microtubule-associated protein 2 (MAP-2) and characterization of additional MAP-2 isoforms. Proc. Natl. Acad. Sci. USA.

[22] Drewes G., Trinczek B., Illenberger S., Biernat J., Schmitt-Ulms G., Meyer H.E., Mandelkow E.M., Mandelkow E. (1995). Microtubule-associated protein/microtubule affinity-regulating kinase (p110mark). A novel protein kinase that regulates tau-microtubule interactions and dynamic instability by phosphorylation at the Alzheimer-specific site serine 262. J. Biol. Chem..

[23] Erickson H.P., Anderson D.E., Osawa M. (2010). FtsZ in bacterial cytokinesis: Cytoskeleton and force generator all in one. Microbiol. Mol. Biol. Rev..

[24] Bi E.F., Lutkenhaus J. (1991). FtsZ ring structure associated with division in Escherichia coli. Nature.

[25] Du S., Lutkenhaus J. (2017). Assembly and activation of the Escherichia coli divisome. Mol. Microbiol..

[26] Bisson-Filho A.W., Hsu Y.P., Squyres G.R., Kuru E., Wu F., Jukes C., Sun Y., Dekker C., Holden S., VanNieuwenhze M.S. (2017). Treadmilling by FtsZ filaments drives peptidoglycan synthesis and bacterial cell division. Science.

[27] Costello M.S., Neumann B., Raimondi M.W., Cuthbert B.J., Holubova J., Garza-Sanchez F., Samad A., Bumba L., Torres J.A., Holznecht N. (2026). Bacteria deliver a microtubule-binding protein into mammalian cells to promote colonization. Science.

[28] Makarova K.S., Yutin N., Bell S.D., Koonin E.V. (2010). Evolution of diverse cell division and vesicle formation systems in Archaea. Nat. Rev. Microbiol..

[29] Spang A., Saw J.H., Jorgensen S.L., Zaremba-Niedzwiedzka K., Martijn J., Lind A.E., van Eijk R., Schleper C., Guy L., Ettema T.J.G. (2015). Complex archaea that bridge the gap between prokaryotes and eukaryotes. Nature.

[30] Wollweber F., Xu J., Ponce-Toledo R.I., Marxer F., Rodrigues-Oliveira T., Possnecker A., Luo Z.H., Malit J.J.L., Kokhanovska A., Wieczorek M. (2025). Microtubules in Asgard archaea. Cell.

[31] Hodges M.E., Scheumann N., Wickstead B., Langdale J.A., Gull K. (2010). Reconstructing the evolutionary history of the centriole from protein components. J. Cell Sci..

[32] Nabais C., Peneda C., Bettencourt-Dias M. (2020). Evolution of centriole assembly. Curr. Biol..

[33] LaFrance B.J., Roostalu J., Henkin G., Greber B.J., Zhang R., Normanno D., McCollum C.O., Surrey T., Nogales E. (2022). Structural transitions in the GTP cap visualized by cryo-electron microscopy of catalytically inactive microtubules. Proc. Natl. Acad. Sci. USA.

[34] Nieuwenhuis J., Brummelkamp T.R. (2019). The Tubulin Detyrosination Cycle: Function and Enzymes. Trends Cell Biol..

[35] Prota A.E., Magiera M.M., Kuijpers M., Bargsten K., Frey D., Wieser M., Jaussi R., Hoogenraad C.C., Kammerer R.A., Janke C. (2013). Structural basis of tubulin tyrosination by tubulin tyrosine ligase. J. Cell Biol..

[36] Szyk A., Deaconescu A.M., Piszczek G., Roll-Mecak A. (2011). Tubulin tyrosine ligase structure reveals adaptation of an ancient fold to bind and modify tubulin. Nat. Struct. Mol. Biol..

[37] van Dijk J., Rogowski K., Miro J., Lacroix B., Edde B., Janke C. (2007). A targeted multienzyme mechanism for selective microtubule polyglutamylation. Mol. Cell.

[38] Janke C., Rogowski K., Wloga D., Regnard C., Kajava A.V., Strub J.M., Temurak N., van Dijk J., Boucher D., van Dorsselaer A. (2005). Tubulin polyglutamylase enzymes are members of the TTL domain protein family. Science.

[39] Garnham C.P., Vemu A., Wilson-Kubalek E.M., Yu I., Szyk A., Lander G.C., Milligan R.A., Roll-Mecak A. (2015). Multivalent Microtubule Recognition by Tubulin Tyrosine Ligase-like Family Glutamylases. Cell.

[40] Garnham C.P., Yu I., Li Y., Roll-Mecak A. (2017). Crystal structure of tubulin tyrosine ligase-like 3 reveals essential architectural elements unique to tubulin monoglycylases. Proc. Natl. Acad. Sci. USA.

[41] Mahalingan K.K., Grotjahn D.A., Li Y., Lander G.C., Zehr E.A., Roll-Mecak A. (2024). Structural basis for alpha-tubulin-specific and modification state-dependent glutamylation. Nat. Chem. Biol..

[42] Rogowski K., Juge F., van Dijk J., Wloga D., Strub J.M., Levilliers N., Thomas D., Bre M.H., Van Dorsselaer A., Gaertig J. (2009). Evolutionary divergence of enzymatic mechanisms for posttranslational polyglycylation. Cell.

[43] Tort O., Tanco S., Rocha C., Bieche I., Seixas C., Bosc C., Andrieux A., Moutin M.J., Aviles F.X., Lorenzo J. (2014). The cytosolic carboxypeptidases CCP2 and CCP3 catalyze posttranslational removal of acidic amino acids. Mol. Biol. Cell.

[44] Berezniuk I., Lyons P.J., Sironi J.J., Xiao H., Setou M., Angeletti R.H., Ikegami K., Fricker L.D. (2013). Cytosolic carboxypeptidase 5 removes alpha- and gamma-linked glutamates from tubulin. J. Biol. Chem..

[45] Rogowski K., van Dijk J., Magiera M.M., Bosc C., Deloulme J.C., Bosson A., Peris L., Gold N.D., Lacroix B., Bosch Grau M. (2010). A family of protein-deglutamylating enzymes associated with neurodegeneration. Cell.

[46] Bosch Grau M., Gonzalez Curto G., Rocha C., Magiera M.M., Marques Sousa P., Giordano T., Spassky N., Janke C. (2013). Tubulin glycylases and glutamylases have distinct functions in stabilization and motility of ependymal cilia. J. Cell Biol..

[47] Wloga D., Webster D.M., Rogowski K., Bre M.H., Levilliers N., Jerka-Dziadosz M., Janke C., Dougan S.T., Gaertig J. (2009). TTLL3 Is a tubulin glycine ligase that regulates the assembly of cilia. Dev. Cell.

[48] Kubo T., Sasaki R., Oda T. (2024). Tubulin glycylation controls ciliary motility through modulation of outer-arm dyneins. Mol. Biol. Cell.

[49] Gadadhar S., Alvarez Viar G., Hansen J.N., Gong A., Kostarev A., Ialy-Radio C., Leboucher S., Whitfield M., Ziyyat A., Toure A. (2021). Tubulin glycylation controls axonemal dynein activity, flagellar beat, and male fertility. Science.

[50] Alvarez Viar G., Klena N., Martino F., Nievergelt A.P., Bolognini D., Capasso P., Pigino G. (2024). Protofilament-specific nanopatterns of tubulin post-translational modifications regulate the mechanics of ciliary beating. Curr. Biol..

[51] Akella J.S., Wloga D., Kim J., Starostina N.G., Lyons-Abbott S., Morrissette N.S., Dougan S.T., Kipreos E.T., Gaertig J. (2010). MEC-17 is an alpha-tubulin acetyltransferase. Nature.

[52] Friedmann D.R., Aguilar A., Fan J., Nachury M.V., Marmorstein R. (2012). Structure of the alpha-tubulin acetyltransferase, alphaTAT1, and implications for tubulin-specific acetylation. Proc. Natl. Acad. Sci. USA.

[53] Kalebic N., Sorrentino S., Perlas E., Bolasco G., Martinez C., Heppenstall P.A. (2013). alphaTAT1 is the major alpha-tubulin acetyltransferase in mice. Nat. Commun..

[54] Coombes C., Yamamoto A., McClellan M., Reid T.A., Plooster M., Luxton G.W., Alper J., Howard J., Gardner M.K. (2016). Mechanism of microtubule lumen entry for the alpha-tubulin acetyltransferase enzyme alphaTAT1. Proc. Natl. Acad. Sci. USA.

[55] Szyk A., Deaconescu A.M., Spector J., Goodman B., Valenstein M.L., Ziolkowska N.E., Kormendi V., Grigorieff N., Roll-Mecak A. (2014). Molecular basis for age-dependent microtubule acetylation by tubulin acetyltransferase. Cell.

[56] Davenport A.M., Collins L.N., Chiu H., Minor P.J., Sternberg P.W., Hoelz A. (2014). Structural and functional characterization of the alpha-tubulin acetyltransferase MEC-17. J. Mol. Biol..

[57] Eshun-Wilson L., Zhang R., Portran D., Nachury M.V., Toso D.B., Lohr T., Vendruscolo M., Bonomi M., Fraser J.S., Nogales E. (2019). Effects of alpha-tubulin acetylation on microtubule structure and stability. Proc. Natl. Acad. Sci. USA.

[58] Hubbert C., Guardiola A., Shao R., Kawaguchi Y., Ito A., Nixon A., Yoshida M., Wang X.F., Yao T.P. (2002). HDAC6 is a microtubule-associated deacetylase. Nature.

[59] Skultetyova L., Ustinova K., Kutil Z., Novakova Z., Pavlicek J., Mikesova J., Trapl D., Baranova P., Havlinova B., Hubalek M. (2017). Human histone deacetylase 6 shows strong preference for tubulin dimers over assembled microtubules. Sci. Rep..

[60] North B.J., Marshall B.L., Borra M.T., Denu J.M., Verdin E. (2003). The human Sir2 ortholog, SIRT2, is an NAD+-dependent tubulin deacetylase. Mol. Cell.

[61] Aillaud C., Bosc C., Peris L., Bosson A., Heemeryck P., Van Dijk J., Le Friec J., Boulan B., Vossier F., Sanman L.E. (2017). Vasohibins/SVBP are tubulin carboxypeptidases (TCPs) that regulate neuron differentiation. Science.

[62] Nieuwenhuis J., Adamopoulos A., Bleijerveld O.B., Mazouzi A., Stickel E., Celie P., Altelaar M., Knipscheer P., Perrakis A., Blomen V.A. (2017). Vasohibins encode tubulin detyrosinating activity. Science.

[63] Barisic M., Silva e Sousa R., Tripathy S.K., Magiera M.M., Zaytsev A.V., Pereira A.L., Janke C., Grishchuk E.L., Maiato H. (2015). Mitosis. Microtubule detyrosination guides chromosomes during mitosis. Science.

[64] Weisbrich A., Honnappa S., Jaussi R., Okhrimenko O., Frey D., Jelesarov I., Akhmanova A., Steinmetz M.O. (2007). Structure-function relationship of CAP-Gly domains. Nat. Struct. Mol. Biol..

[65] Bieling P., Kandels-Lewis S., Telley I.A., van Dijk J., Janke C., Surrey T. (2008). CLIP-170 tracks growing microtubule ends by dynamically recognizing composite EB1/tubulin-binding sites. J. Cell Biol..

[66] Fan X., McKenney R.J. (2023). Control of motor landing and processivity by the CAP-Gly domain in the KIF13B tail. Nat. Commun..

[67] Hammond J.W., Huang C.F., Kaech S., Jacobson C., Banker G., Verhey K.J. (2010). Posttranslational modifications of tubulin and the polarized transport of kinesin-1 in neurons. Mol. Biol. Cell.

[68] Kaul N., Soppina V., Verhey K.J. (2014). Effects of alpha-tubulin K40 acetylation and detyrosination on kinesin-1 motility in a purified system. Biophys. J..

[69] Lacroix B., van Dijk J., Gold N.D., Guizetti J., Aldrian-Herrada G., Rogowski K., Gerlich D.W., Janke C. (2010). Tubulin polyglutamylation stimulates spastin-mediated microtubule severing. J. Cell Biol..

[70] Han H., Schubert H.L., McCullough J., Monroe N., Purdy M.D., Yeager M., Sundquist W.I., Hill C.P. (2020). Structure of spastin bound to a glutamate-rich peptide implies a hand-over-hand mechanism of substrate translocation. J. Biol. Chem..

[71] Valenstein M.L., Roll-Mecak A. (2016). Graded Control of Microtubule Severing by Tubulin Glutamylation. Cell.

[72] Patir A., Fraser A.M., Barnett M.W., McTeir L., Rainger J., Davey M.G., Freeman T.C. (2020). The transcriptional signature associated with human motile cilia. Sci. Rep..

[73] Rayamajhi D., Ege M., Ukhanov K., Ringers C., Zhang Y., Jung I., D’Gama P.P., Li S.S., Cosacak M.I., Kizil C. (2024). The forkhead transcription factor Foxj1 controls vertebrate olfactory cilia biogenesis and sensory neuron differentiation. PLoS Biol..

[74] Choksi S.P., Lauter G., Swoboda P., Roy S. (2014). Switching on cilia: Transcriptional networks regulating ciliogenesis. Development.

[75] Miller J.G., Liu Y., Williams C.W., Smith H.E., O’Connell K.F. (2016). The E2F-DP1 Transcription Factor Complex Regulates Centriole Duplication in Caenorhabditis elegans. G3.

[76] Nigg E.A., Stearns T. (2011). The centrosome cycle: Centriole biogenesis, duplication and inherent asymmetries. Nat. Cell Biol..

[77] Nigg E.A., Holland A.J. (2018). Once and only once: Mechanisms of centriole duplication and their deregulation in disease. Nat. Rev. Mol. Cell Biol..

[78] Breslow D.K., Holland A.J. (2019). Mechanism and Regulation of Centriole and Cilium Biogenesis. Annu. Rev. Biochem..

[79] Woodruff J.B., Wueseke O., Hyman A.A. (2014). Pericentriolar material structure and dynamics. Philos. Trans. R. Soc. Lond. B Biol. Sci..

[80] Ma D., Wang F., Teng J., Huang N., Chen J. (2023). Structure and function of distal and subdistal appendages of the mother centriole. J. Cell Sci..

[81] Nakazawa Y., Hiraki M., Kamiya R., Hirono M. (2007). SAS-6 is a cartwheel protein that establishes the 9-fold symmetry of the centriole. Curr. Biol..

[82] Kitagawa D., Vakonakis I., Olieric N., Hilbert M., Keller D., Olieric V., Bortfeld M., Erat M.C., Fluckiger I., Gonczy P. (2011). Structural basis of the 9-fold symmetry of centrioles. Cell.

[83] van Breugel M., Hirono M., Andreeva A., Yanagisawa H.A., Yamaguchi S., Nakazawa Y., Morgner N., Petrovich M., Ebong I.O., Robinson C.V. (2011). Structures of SAS-6 suggest its organization in centrioles. Science.

[84] Lin Y.C., Chang C.W., Hsu W.B., Tang C.J., Lin Y.N., Chou E.J., Wu C.T., Tang T.K. (2013). Human microcephaly protein CEP135 binds to hSAS-6 and CPAP, and is required for centriole assembly. EMBO J..

[85] Vulprecht J., David A., Tibelius A., Castiel A., Konotop G., Liu F., Bestvater F., Raab M.S., Zentgraf H., Izraeli S. (2012). STIL is required for centriole duplication in human cells. J. Cell Sci..

[86] Comartin D., Gupta G.D., Fussner E., Coyaud E., Hasegan M., Archinti M., Cheung S.W., Pinchev D., Lawo S., Raught B. (2013). CEP120 and SPICE1 cooperate with CPAP in centriole elongation. Curr. Biol..

[87] Archinti M., Lacasa C., Teixido-Travesa N., Luders J. (2010). SPICE--a previously uncharacterized protein required for centriole duplication and mitotic chromosome congression. J. Cell Sci..

[88] Chen H.Y., Wu C.T., Tang C.C., Lin Y.N., Wang W.J., Tang T.K. (2017). Human microcephaly protein RTTN interacts with STIL and is required to build full-length centrioles. Nat. Commun..

[89] Chang C.W., Hsu W.B., Tsai J.J., Tang C.J., Tang T.K. (2016). CEP295 interacts with microtubules and is required for centriole elongation. J. Cell Sci..

[90] Fong C.S., Kim M., Yang T.T., Liao J.C., Tsou M.F. (2014). SAS-6 assembly templated by the lumen of cartwheel-less centrioles precedes centriole duplication. Dev. Cell.

[91] Klena N., Le Guennec M., Tassin A.M., van den Hoek H., Erdmann P.S., Schaffer M., Geimer S., Aeschlimann G., Kovacik L., Sadian Y. (2020). Architecture of the centriole cartwheel-containing region revealed by cryo-electron tomography. EMBO J..

[92] Guichard P., Desfosses A., Maheshwari A., Hachet V., Dietrich C., Brune A., Ishikawa T., Sachse C., Gonczy P. (2012). Cartwheel architecture of Trichonympha basal body. Science.

[93] Kantsadi A.L., Hatzopoulos G.N., Gonczy P., Vakonakis I. (2022). Structures of SAS-6 coiled coil hold implications for the polarity of the centriolar cartwheel. Structure.

[94] Cai B., Xu J., Collet E.H., Aarts E., Luo L., Leitner A., Ishikawa T., Beltrao P., Pearson C.G., Pilhofer M. (2025). Structure and assembly of the A-C linker connecting microtubule triplets in centrioles. Sci. Adv..

[95] Bournonville L., Laporte M.H., Borgers S., Guichard P., Hamel V. (2025). The A-C linker controls centriole structural integrity and duplication. Nat. Commun..

[96] Laporte M.H., Gambarotto D., Bertiaux E., Bournonville L., Louvel V., Nunes J.M., Borgers S., Hamel V., Guichard P. (2024). Time-series reconstruction of the molecular architecture of human centriole assembly. Cell.

[97] Tanos B.E., Yang H.J., Soni R., Wang W.J., Macaluso F.P., Asara J.M., Tsou M.F. (2013). Centriole distal appendages promote membrane docking, leading to cilia initiation. Genes. Dev..

[98] Graser S., Stierhof Y.D., Lavoie S.B., Gassner O.S., Lamla S., Le Clech M., Nigg E.A. (2007). Cep164, a novel centriole appendage protein required for primary cilium formation. J. Cell Biol..

[99] Uzbekov R., Alieva I. (2018). Who are you, subdistal appendages of centriole?. Open Biol..

[100] Sala C., Wurtz M., Atorino E.S., Neuner A., Partscht P., Hoffmann T., Eustermann S., Schiebel E. (2024). An interaction network of inner centriole proteins organised by POC1A-POC1B heterodimer crosslinks ensures centriolar integrity. Nat. Commun..

[101] Arslanhan M.D., Cengiz-Emek S., Odabasi E., Steib E., Hamel V., Guichard P., Firat-Karalar E.N. (2023). CCDC15 localizes to the centriole inner scaffold and controls centriole length and integrity. J. Cell Biol..

[102] Gilliam J.C., Chang J.T., Sandoval I.M., Zhang Y., Li T., Pittler S.J., Chiu W., Wensel T.G. (2012). Three-dimensional architecture of the rod sensory cilium and its disruption in retinal neurodegeneration. Cell.

[103] Fais D.A., Nadezhdina E.S., Chentsov Y.S. (1986). The centriolar rim. The structure that maintains the configuration of centrioles and basal bodies in the absence of their microtubules. Exp. Cell Res..

[104] Steib E., Laporte M.H., Gambarotto D., Olieric N., Zheng C., Borgers S., Olieric V., Le Guennec M., Koll F., Tassin A.M. (2020). WDR90 is a centriolar microtubule wall protein important for centriole architecture integrity. eLife.

[105] Takeda Y., Chinen T., Honda S., Takatori S., Okuda S., Yamamoto S., Fukuyama M., Takeuchi K., Tomita T., Hata S. (2024). Molecular basis promoting centriole triplet microtubule assembly. Nat. Commun..

[106] Pearson C.G., Winey M. (2009). Basal body assembly in ciliates: The power of numbers. Traffic.

[107] Li S., Fernandez J.J., Marshall W.F., Agard D.A. (2012). Three-dimensional structure of basal body triplet revealed by electron cryo-tomography. EMBO J..

[108] Ichikawa M., Liu D., Kastritis P.L., Basu K., Hsu T.C., Yang S., Bui K.H. (2017). Subnanometre-resolution structure of the doublet microtubule reveals new classes of microtubule-associated proteins. Nat. Commun..

[109] Ma S., Li L., Li Z., Luo S., Liu Q., Du W., Qiu B., Gui M., Zhu X., Guo Q. (2025). In situ cryo-electron tomography reveals the progressive biogenesis of basal bodies and cilia in mouse ependymal cells. Nat. Commun..

[110] Ma M., Stoyanova M., Rademacher G., Dutcher S.K., Brown A., Zhang R. (2019). Structure of the Decorated Ciliary Doublet Microtubule. Cell.

[111] Walton T., Gui M., Velkova S., Fassad M.R., Hirst R.A., Haarman E., O’Callaghan C., Bottier M., Burgoyne T., Mitchison H.M. (2023). Axonemal structures reveal mechanoregulatory and disease mechanisms. Nature.

[112] Gui M., Croft J.T., Zabeo D., Acharya V., Kollman J.M., Burgoyne T., Hoog J.L., Brown A. (2022). SPACA9 is a lumenal protein of human ciliary singlet and doublet microtubules. Proc. Natl. Acad. Sci. USA.

[113] Gui M., Wang X., Dutcher S.K., Brown A., Zhang R. (2022). Ciliary central apparatus structure reveals mechanisms of microtubule patterning. Nat. Struct. Mol. Biol..

[114] Gui M., Farley H., Anujan P., Anderson J.R., Maxwell D.W., Whitchurch J.B., Botsch J.J., Qiu T., Meleppattu S., Singh S.K. (2021). De novo identification of mammalian ciliary motility proteins using cryo-EM. Cell.

[115] Han L., Rao Q., Yang R., Wang Y., Chai P., Xiong Y., Zhang K. (2022). Cryo-EM structure of an active central apparatus. Nat. Struct. Mol. Biol..

[116] Loreng T.D., Smith E.F. (2017). The Central Apparatus of Cilia and Eukaryotic Flagella. Cold Spring Harb. Perspect. Biol..

[117] Khalifa A.A.Z., Ichikawa M., Dai D., Kubo S., Black C.S., Peri K., McAlear T.S., Veyron S., Yang S.K., Vargas J. (2020). The inner junction complex of the cilia is an interaction hub that involves tubulin post-translational modifications. eLife.

[118] Leung M.R., Sun C., Zeng J., Anderson J.R., Niu Q., Huang W., Noteborn W.E.M., Brown A., Zeev-Ben-Mordehai T., Zhang R. (2025). Structural diversity of axonemes across mammalian motile cilia. Nature.

[119] Takeda Y., Kajikawa E., Wang J., Ishida M., Alsheimer M., Shibuya H. (2025). Centrin-POC5 inner scaffold provides distal centriole integrity for sperm flagellar assembly. Sci. Adv..

[120] Le Guennec M., Klena N., Gambarotto D., Laporte M.H., Tassin A.M., van den Hoek H., Erdmann P.S., Schaffer M., Kovacik L., Borgers S. (2020). A helical inner scaffold provides a structural basis for centriole cohesion. Sci. Adv..

[121] Mercey O., Kostic C., Bertiaux E., Giroud A., Sadian Y., Gaboriau D.C.A., Morrison C.G., Chang N., Arsenijevic Y., Guichard P. (2022). The connecting cilium inner scaffold provides a structural foundation that protects against retinal degeneration. PLoS Biol..

[122] Weisz Hubshman M., Broekman S., van Wijk E., Cremers F., Abu-Diab A., Khateb S., Tzur S., Lagovsky I., Smirin-Yosef P., Sharon D. (2018). Whole-exome sequencing reveals POC5 as a novel gene associated with autosomal recessive retinitis pigmentosa. Hum. Mol. Genet..

[123] Avidor-Reiss T. (2024). Renaissance in sperm cytoplasmic contribution to infertility. J. Assist. Reprod. Genet..

[124] Zheng J., Liu H., Zhu L., Chen Y., Zhao H., Zhang W., Li F., Xie L., Yan X., Zhu X. (2019). Microtubule-bundling protein Spef1 enables mammalian ciliary central apparatus formation. J. Mol. Cell Biol..

[125] Guha M., Vasudevan K.K., Jiang Y.Y., Louka P., Sharma N., Parra M., Lechtreck K.F., Tomasi R.F., Baroud C.N., Dupuis-Williams P. (2025). SPEF1 mediates assembly of the central pair microtubule complexes in cilia of Tetrahymena. bioRxiv.

[126] Orosz F. (2021). On the TPPP-like proteins of flagellated fungi. Fungal Biol..

[127] Orosz F. (2012). A new protein superfamily: TPPP-like proteins. PLoS ONE.

[128] Ponting C.P. (2006). A novel domain suggests a ciliary function for ASPM, a brain size determining gene. Bioinformatics.

[129] Schou K.B., Morthorst S.K., Christensen S.T., Pedersen L.B. (2014). Identification of conserved, centrosome-targeting ASH domains in TRAPPII complex subunits and TRAPPC8. Cilia.

[130] Zhao L., Hou Y., McNeill N.A., Witman G.B. (2020). The unity and diversity of the ciliary central apparatus. Philos. Trans. R. Soc. Lond. B Biol. Sci..

[131] Desai S., D’Souza J.S. (2025). ASH: A new protein domain on the horizon. J. Proteins Proteom..

[132] Kumar D., Reiter J. (2021). How the centriole builds its cilium: Of mothers, daughters, and the acquisition of appendages. Curr. Opin. Struct. Biol..

[133] Wallmeier J., Nielsen K.G., Kuehni C.E., Lucas J.S., Leigh M.W., Zariwala M.A., Omran H. (2020). Motile ciliopathies. Nat. Rev. Dis. Primers.

[134] Hoffmann I. (2021). Centrosomes in mitotic spindle assembly and orientation. Curr. Opin. Struct. Biol..

[135] Cassimeris L. (1999). Accessory protein regulation of microtubule dynamics throughout the cell cycle. Curr. Opin. Cell Biol..

[136] Kraus J., Alfaro-Aco R., Gouveia B., Petry S. (2023). Microtubule nucleation for spindle assembly: One molecule at a time. Trends Biochem. Sci..

[137] Biven E., Wang J.T. (2025). Mechanisms underlying centriole stability. J. Biol. Chem..

[138] Peterman E.J., Scholey J.M. (2009). Mitotic microtubule crosslinkers: Insights from mechanistic studies. Curr. Biol..

[139] Gruss O.J., Wittmann M., Yokoyama H., Pepperkok R., Kufer T., Sillje H., Karsenti E., Mattaj I.W., Vernos I. (2002). Chromosome-induced microtubule assembly mediated by TPX2 is required for spindle formation in HeLa cells. Nat. Cell Biol..

[140] Schatz C.A., Santarella R., Hoenger A., Karsenti E., Mattaj I.W., Gruss O.J., Carazo-Salas R.E. (2003). Importin alpha-regulated nucleation of microtubules by TPX2. EMBO J..

[141] Brunet S., Sardon T., Zimmerman T., Wittmann T., Pepperkok R., Karsenti E., Vernos I. (2004). Characterization of the TPX2 domains involved in microtubule nucleation and spindle assembly in Xenopus egg extracts. Mol. Biol. Cell.

[142] Liang Z., Huang J., Wang Y., Hua S., Jiang K. (2025). Diverse microtubule-binding repeats regulate TPX2 activities at distinct locations within the spindle. J. Cell Biol..

[143] Sillje H.H., Nagel S., Korner R., Nigg E.A. (2006). HURP is a Ran-importin beta-regulated protein that stabilizes kinetochore microtubules in the vicinity of chromosomes. Curr. Biol..

[144] Wong J., Fang G. (2006). HURP controls spindle dynamics to promote proper interkinetochore tension and efficient kinetochore capture. J. Cell Biol..

[145] Zhang Y., Tan L., Yang Q., Li C., Liou Y.C. (2018). The microtubule-associated protein HURP recruits the centrosomal protein TACC3 to regulate K-fiber formation and support chromosome congression. J. Biol. Chem..

[146] Valdez V.A., Ma M., Gouveia B., Zhang R., Petry S. (2024). HURP facilitates spindle assembly by stabilizing microtubules and working synergistically with TPX2. Nat. Commun..

[147] Booth D.G., Hood F.E., Prior I.A., Royle S.J. (2011). A TACC3/ch-TOG/clathrin complex stabilises kinetochore fibres by inter-microtubule bridging. EMBO J..

[148] Hood F.E., Williams S.J., Burgess S.G., Richards M.W., Roth D., Straube A., Pfuhl M., Bayliss R., Royle S.J. (2013). Coordination of adjacent domains mediates TACC3-ch-TOG-clathrin assembly and mitotic spindle binding. J. Cell Biol..

[149] Widlund P.O., Stear J.H., Pozniakovsky A., Zanic M., Reber S., Brouhard G.J., Hyman A.A., Howard J. (2011). XMAP215 polymerase activity is built by combining multiple tubulin-binding TOG domains and a basic lattice-binding region. Proc. Natl. Acad. Sci. USA.

[150] Dionne M.A., Howard L., Compton D.A. (1999). NuMA is a component of an insoluble matrix at mitotic spindle poles. Cell Motil. Cytoskelet..

[151] Merdes A., Heald R., Samejima K., Earnshaw W.C., Cleveland D.W. (2000). Formation of spindle poles by dynein/dynactin-dependent transport of NuMA. J. Cell Biol..

[152] Hueschen C.L., Kenny S.J., Xu K., Dumont S. (2017). NuMA recruits dynein activity to microtubule minus-ends at mitosis. eLife.

[153] Cho N.H., Aslan M., Taheri A., Yildiz A., Dumont S. (2025). NuMA mechanically reinforces the spindle independently of its partner dynein. Curr. Biol..

[154] Margolis R.L., Rauch C.T., Pirollet F., Job D. (1990). Specific association of STOP protein with microtubules in vitro and with stable microtubules in mitotic spindles of cultured cells. EMBO J..

[155] Lefevre J., Savarin P., Gans P., Hamon L., Clement M.J., David M.O., Bosc C., Andrieux A., Curmi P.A. (2013). Structural basis for the association of MAP6 protein with microtubules and its regulation by calmodulin. J. Biol. Chem..

[156] Ibi M., Zou P., Inoko A., Shiromizu T., Matsuyama M., Hayashi Y., Enomoto M., Mori D., Hirotsune S., Kiyono T. (2011). Trichoplein controls microtubule anchoring at the centrosome by binding to Odf2 and ninein. J. Cell Sci..

[157] Lauriola A., Martello A., Fantini S., Marverti G., Zanocco-Marani T., Davalli P., Guardavaccaro D., Mai S., Caporali A., D’Arca D. (2020). Depletion of Trichoplein (TpMs) Causes Chromosome Mis-Segregation, DNA Damage and Chromosome Instability in Cancer Cells. Cancers.

[158] Liu Q., Ruderman J.V. (2006). Aurora A, mitotic entry, and spindle bipolarity. Proc. Natl. Acad. Sci. USA.

[159] Roghi C., Giet R., Uzbekov R., Morin N., Chartrain I., Le Guellec R., Couturier A., Doree M., Philippe M., Prigent C. (1998). The Xenopus protein kinase pEg2 associates with the centrosome in a cell cycle-dependent manner, binds to the spindle microtubules and is involved in bipolar mitotic spindle assembly. J. Cell Sci..

[160] Kufer T.A., Sillje H.H., Korner R., Gruss O.J., Meraldi P., Nigg E.A. (2002). Human TPX2 is required for targeting Aurora-A kinase to the spindle. J. Cell Biol..

[161] Bayliss R., Sardon T., Vernos I., Conti E. (2003). Structural basis of Aurora-A activation by TPX2 at the mitotic spindle. Mol. Cell.

[162] Inoko A., Matsuyama M., Goto H., Ohmuro-Matsuyama Y., Hayashi Y., Enomoto M., Ibi M., Urano T., Yonemura S., Kiyono T. (2012). Trichoplein and Aurora A block aberrant primary cilia assembly in proliferating cells. J. Cell Biol..

[163] Zheng Y., Wong M.L., Alberts B., Mitchison T. (1995). Nucleation of microtubule assembly by a gamma-tubulin-containing ring complex. Nature.

[164] Moritz M., Braunfeld M.B., Sedat J.W., Alberts B., Agard D.A. (1995). Microtubule nucleation by gamma-tubulin-containing rings in the centrosome. Nature.

[165] Liu P., Würtz M., Zupa E., Pfeffer S., Schiebel E. (2021). Microtubule nucleation: The waltz between γ-tubulin ring complex and associated proteins. Curr. Opin. Cell Biol..

[166] Wiese C., Zheng Y. (2000). A new function for the gamma-tubulin ring complex as a microtubule minus-end cap. Nat. Cell Biol..

[167] Dendooven T., Yatskevich S., Burt A., Chen Z.A., Bellini D., Rappsilber J., Kilmartin J.V., Barford D. (2024). Structure of the native gamma-tubulin ring complex capping spindle microtubules. Nat. Struct. Mol. Biol..

[168] Lin T.C., Neuner A., Schiebel E. (2015). Targeting of gamma-tubulin complexes to microtubule organizing centers: Conservation and divergence. Trends Cell Biol..

[169] Haren L., Remy M.H., Bazin I., Callebaut I., Wright M., Merdes A. (2006). NEDD1-dependent recruitment of the gamma-tubulin ring complex to the centrosome is necessary for centriole duplication and spindle assembly. J. Cell Biol..

[170] Choi Y.K., Liu P., Sze S.K., Dai C., Qi R.Z. (2010). CDK5RAP2 stimulates microtubule nucleation by the gamma-tubulin ring complex. J. Cell Biol..

[171] Fong K.W., Choi Y.K., Rattner J.B., Qi R.Z. (2008). CDK5RAP2 Is a Pericentriolar Protein That Functions in Centrosomal Attachment of the Gamma-Tubulin Ring Complex. Mol. Biol. Cell.

[172] Lin T.C., Neuner A., Schlosser Y.T., Scharf A.N., Weber L., Schiebel E. (2014). Cell-cycle dependent phosphorylation of yeast pericentrin regulates gamma-TuSC-mediated microtubule nucleation. eLife.

[173] Serna M., Zimmermann F., Vineethakumari C., Gonzalez-Rodriguez N., Llorca O., Luders J. (2024). CDK5RAP2 activates microtubule nucleator gammaTuRC by facilitating template formation and actin release. Dev. Cell.

[174] Brilot A.F., Lyon A.S., Zelter A., Viswanath S., Maxwell A., MacCoss M.J., Muller E.G., Sali A., Davis T.N., Agard D.A. (2021). CM1-driven assembly and activation of yeast γ-tubulin small complex underlies microtubule nucleation. eLife.

[175] Uehara R., Nozawa R.S., Tomioka A., Petry S., Vale R.D., Obuse C., Goshima G. (2009). The augmin complex plays a critical role in spindle microtubule generation for mitotic progression and cytokinesis in human cells. Proc. Natl. Acad. Sci. USA.

[176] Petry S., Groen A.C., Ishihara K., Mitchison T.J., Vale R.D. (2013). Branching microtubule nucleation in Xenopus egg extracts mediated by augmin and TPX2. Cell.

[177] Song J.G., King M.R., Zhang R., Kadzik R.S., Thawani A., Petry S. (2018). Mechanism of how augmin directly targets the gamma-tubulin ring complex to microtubules. J. Cell Biol..

[178] Wieczorek M., Urnavicius L., Ti S.C., Molloy K.R., Chait B.T., Kapoor T.M. (2020). Asymmetric Molecular Architecture of the Human Gamma-Tubulin Ring Complex. Cell.

[179] Consolati T., Locke J., Roostalu J., Chen Z.A., Gannon J., Asthana J., Lim W.M., Martino F., Cvetkovic M.A., Rappsilber J. (2020). Microtubule Nucleation Properties of Single Human gammaTuRCs Explained by Their Cryo-EM Structure. Dev. Cell.

[180] Aher A., Urnavicius L., Xue A., Neselu K., Kapoor T.M. (2024). Structure of the gamma-tubulin ring complex-capped microtubule. Nat. Struct. Mol. Biol..

[181] Xu Y., Munoz-Hernandez H., Krutyholowa R., Marxer F., Cetin F., Wieczorek M. (2024). Partial closure of the gamma-tubulin ring complex by CDK5RAP2 activates microtubule nucleation. Dev. Cell.

[182] Zupa E., Wurtz M., Neuner A., Hoffmann T., Rettel M., Bohler A., Vermeulen B.J.A., Eustermann S., Schiebel E., Pfeffer S. (2022). The augmin complex architecture reveals structural insights into microtubule branching. Nat. Commun..

[183] Wurtz M., Tonon G., Vermeulen B.J.A., Zezlina M., Gao Q., Neuner A., Seidl A., Konig M., Harkenthal M., Eustermann S. (2025). Conserved function of the HAUS6 calponin homology domain in anchoring augmin for microtubule branching. Nat. Commun..

[184] Travis S.M., Mahon B.P., Huang W., Ma M., Rale M.J., Kraus J., Taylor D.J., Zhang R., Petry S. (2023). Integrated model of the vertebrate augmin complex. Nat. Commun..

[185] Sanchez A.D., Feldman J.L. (2017). Microtubule-organizing centers: From the centrosome to non-centrosomal sites. Curr. Opin. Cell..

[186] Schweizer N., Haren L., Dutto I., Viais R., Lacasa C., Merdes A., Luders J. (2021). Sub-centrosomal mapping identifies augmin-γTuRC as part of a centriole-stabilizing scaffold. Nat. Commun..

[187] Gao Q., Hofer F.W., Filbeck S., Vermeulen B.J.A., Wurtz M., Neuner A., Kaplan C., Zezlina M., Sala C., Shin H. (2025). Structural mechanisms for centrosomal recruitment and organization of the microtubule nucleator gamma-TuRC. Nat. Commun..

[188] Piehl M., Cassimeris L. (2003). Organization and dynamics of growing microtubule plus ends during early mitosis. Mol. Biol. Cell.

[189] Diamantopoulos G.S., Perez F., Goodson H.V., Batelier G., Melki R., Kreis T.E., Rickard J.E. (1999). Dynamic localization of CLIP-170 to microtubule plus ends is coupled to microtubule assembly. J. Cell Biol..

[190] Mimori-Kiyosue Y., Grigoriev I., Lansbergen G., Sasaki H., Matsui C., Severin F., Galjart N., Grosveld F., Vorobjev I., Tsukita S. (2005). CLASP1 and CLASP2 bind to EB1 and regulate microtubule plus-end dynamics at the cell cortex. J. Cell Biol..

[191] Patel K., Nogales E., Heald R. (2012). Multiple domains of human CLASP contribute to microtubule dynamics and organization in vitro and in Xenopus egg extracts. Cytoskeleton.

[192] Powers A.F., Franck A.D., Gestaut D.R., Cooper J., Gracyzk B., Wei R.R., Wordeman L., Davis T.N., Asbury C.L. (2009). The Ndc80 kinetochore complex forms load-bearing attachments to dynamic microtubule tips via biased diffusion. Cell.

[193] Akiyoshi B., Sarangapani K.K., Powers A.F., Nelson C.R., Reichow S.L., Arellano-Santoyo H., Gonen T., Ranish J.A., Asbury C.L., Biggins S. (2010). Tension directly stabilizes reconstituted kinetochore-microtubule attachments. Nature.

[194] Tanaka K., Mukae N., Dewar H., van Breugel M., James E.K., Prescott A.R., Antony C., Tanaka T.U. (2005). Molecular mechanisms of kinetochore capture by spindle microtubules. Nature.

[195] Holy T.E., Leibler S. (1994). Dynamic instability of microtubules as an efficient way to search in space. Proc. Natl. Acad. Sci. USA.

[196] Hayden J.H., Bowser S.S., Rieder C.L. (1990). Kinetochores capture astral microtubules during chromosome attachment to the mitotic spindle: Direct visualization in live newt lung cells. J. Cell Biol..

[197] Rogers S.L., Rogers G.C., Sharp D.J., Vale R.D. (2002). Drosophila EB1 is important for proper assembly, dynamics, and positioning of the mitotic spindle. J. Cell Biol..

[198] Green R.A., Wollman R., Kaplan K.B. (2005). APC and EB1 function together in mitosis to regulate spindle dynamics and chromosome alignment. Mol. Biol. Cell.

[199] Maiato H., Fairley E.A.L., Rieder C.L., Swedlow J.R., Sunkel C.E., Earnshaw W.C. (2003). Human CLASP1 is an outer kinetochore component that regulates spindle microtubule dynamics. Cell.

[200] Tanenbaum M.E., Galjart N., van Vugt M., Medema R.H. (2006). CLIP-170 facilitates the formation of kinetochore-microtubule attachments. EMBO J..

[201] Maffini S., Maia A.R., Manning A.L., Maliga Z., Pereira A.L., Junqueira M., Shevchenko A., Hyman A., Yates J.R., Galjart N. (2009). Motor-independent targeting of CLASPs to kinetochores by CENP-E promotes microtubule turnover and poleward flux. Curr. Biol..

[202] Liu J., Wang Z., Jiang K., Zhang L., Zhao L., Hua S., Yan F., Yang Y., Wang D., Fu C. (2009). PRC1 cooperates with CLASP1 to organize central spindle plasticity in mitosis. J. Biol. Chem..

[203] Maia A.R., Garcia Z., Kabeche L., Barisic M., Maffini S., Macedo-Ribeiro S., Cheeseman I.M., Compton D.A., Kaverina I., Maiato H. (2012). Cdk1 and Plk1 mediate a CLASP2 phospho-switch that stabilizes kinetochore-microtubule attachments. J. Cell Biol..

[204] Mimori-Kiyosue Y., Grigoriev I., Sasaki H., Matsui C., Akhmanova A., Tsukita S., Vorobjev I. (2006). Mammalian CLASPs are required for mitotic spindle organization and kinetochore alignment. Genes. Cells.

[205] Pereira A.L., Pereira A.J., Maia A.R., Drabek K., Sayas C.L., Hergert P.J., Lince-Faria M., Matos I., Duque C., Stepanova T. (2006). Mammalian CLASP1 and CLASP2 cooperate to ensure mitotic fidelity by regulating spindle and kinetochore function. Mol. Biol. Cell.

[206] Maiato H., Sampaio P., Lemos C.L., Findlay J., Carmena M., Earnshaw W.C., Sunkel C.E. (2002). MAST/Orbit has a role in microtubule-kinetochore attachment and is essential for chromosome alignment and maintenance of spindle bipolarity. J. Cell Biol..

[207] Inoue Y.H., Savoian M.S., Suzuki T., Máthé E., Yamamoto M.T., Glover D.M. (2004). Mutations in orbit/mast reveal that the central spindle is comprised of two microtubule populations, those that initiate cleavage and those that propagate furrow ingression. J. Cell Biol..

[208] Hayashi I., Ikura M. (2003). Crystal structure of the amino-terminal microtubule-binding domain of end-binding protein 1 (EB1). J. Biol. Chem..

[209] Maurer S.P., Fourniol F.J., Bohner G., Moores C.A., Surrey T. (2012). EBs recognize a nucleotide-dependent structural cap at growing microtubule ends. Cell.

[210] Ayaz P., Ye X., Huddleston P., Brautigam C.A., Rice L.M. (2012). A TOG:alphabeta-tubulin complex structure reveals conformation-based mechanisms for a microtubule polymerase. Science.

[211] Aher A., Kok M., Sharma A., Rai A., Olieric N., Rodriguez-Garcia R., Katrukha E.A., Weinert T., Olieric V., Kapitein L.C. (2018). CLASP Suppresses Microtubule Catastrophes through a Single TOG Domain. Dev. Cell.

[212] Honnappa S., Gouveia S.M., Weisbrich A., Damberger F.F., Bhavesh N.S., Jawhari H., Grigoriev I., van Rijssel F.J., Buey R.M., Lawera A. (2009). An EB1-binding motif acts as a microtubule tip localization signal. Cell.

[213] Ciferri C., Pasqualato S., Screpanti E., Varetti G., Santaguida S., Dos Reis G., Maiolica A., Polka J., De Luca J.G., De Wulf P. (2008). Implications for kinetochore-microtubule attachment from the structure of an engineered Ndc80 complex. Cell.

[214] Alushin G.M., Musinipally V., Matson D., Tooley J., Stukenberg P.T., Nogales E. (2012). Multimodal microtubule binding by the Ndc80 kinetochore complex. Nat. Struct. Mol. Biol..

[215] Volkov V.A., Huis In ‘t Veld P.J., Dogterom M., Musacchio A. (2018). Multivalency of NDC80 in the outer kinetochore is essential to track shortening microtubules and generate forces. eLife.

[216] Wei R.R., Al-Bassam J., Harrison S.C. (2007). The Ndc80/HEC1 complex is a contact point for kinetochore-microtubule attachment. Nat. Struct. Mol. Biol..

[217] Sundin L.J., Guimaraes G.J., Deluca J.G. (2011). The NDC80 complex proteins Nuf2 and Hec1 make distinct contributions to kinetochore-microtubule attachment in mitosis. Mol. Biol. Cell.

[218] Alushin G.M., Ramey V.H., Pasqualato S., Ball D.A., Grigorieff N., Musacchio A., Nogales E. (2010). The Ndc80 kinetochore complex forms oligomeric arrays along microtubules. Nature.

[219] Helgeson L.A., Zelter A., Riffle M., MacCoss M.J., Asbury C.L., Davis T.N. (2018). Human Ska complex and Ndc80 complex interact to form a load-bearing assembly that strengthens kinetochore-microtubule attachments. Proc. Natl. Acad. Sci. USA.

[220] Huis In ‘t Veld P.J., Volkov V.A., Stender I.D., Musacchio A., Dogterom M. (2019). Molecular determinants of the Ska-Ndc80 interaction and their influence on microtubule tracking and force-coupling. eLife.

[221] Welburn J.P.I., Grishchuk E.L., Backer C.B., Wilson-Kubalek E.M., Yates J.R., Cheeseman I.M. (2009). The human kinetochore Ska1 complex facilitates microtubule depolymerization-coupled motility. Dev. Cell.

[222] Schmidt J.C., Arthanari H., Boeszoermenyi A., Dashkevich N.M., Wilson-Kubalek E.M., Monnier N., Markus M., Oberer M., Milligan R.A., Bathe M. (2012). The kinetochore-bound Ska1 complex tracks depolymerizing microtubules and binds to curved protofilaments. Dev. Cell.

[223] Janczyk P.L., Skorupka K.A., Tooley J.G., Matson D.R., Kestner C.A., West T., Pornillos O., Stukenberg P.T. (2017). Mechanism of Ska recruitment by Ndc80 complexes to kinetochores. Dev. Cell.

[224] Mollinari C., Kleman J.P., Jiang W., Schoehn G., Hunter T., Margolis R.L. (2002). PRC1 is a microtubule binding and bundling protein essential to maintain the mitotic spindle midzone. J. Cell Biol..

[225] Kajtez J., Solomatina A., Novak M., Polak B., Vukusic K., Rudiger J., Cojoc G., Milas A., Sumanovac Sestak I., Risteski P. (2016). Overlap microtubules link sister k-fibres and balance the forces on bi-oriented kinetochores. Nat. Commun..

[226] Matkovic J., Ghosh S., Cosic M., Eibes S., Barisic M., Pavin N., Tolic I.M. (2022). Kinetochore- and chromosome-driven transition of microtubules into bundles promotes spindle assembly. Nat. Commun..

[227] Jagric M., Risteski P., Martincic J., Milas A., Tolic I.M. (2021). Optogenetic control of PRC1 reveals its role in chromosome alignment on the spindle by overlap length-dependent forces. eLife.

[228] Kikuchi K., Sakamoto Y., Uezu A., Yamamoto H., Ishiguro K.I., Shimamura K., Saito T., Hisanaga S.I., Nakanishi H. (2022). Map7D2 and Map7D1 facilitate microtubule stabilization through distinct mechanisms in neuronal cells. Life Sci. Alliance.

[229] Dullovi A., Ozgencil M., Rajvee V., Tse W.Y., Cutillas P.R., Martin S.A., Horejsi Z. (2023). Microtubule-associated proteins MAP7 and MAP7D1 promote DNA double-strand break repair in the G1 cell cycle phase. iScience.

[230] Kucukvardar S., Karabay A. (2025). A novel MAP7D1 mutation causes mitotic defects and RPS14 accumulation in Shwachman-Diamond syndrome patient cells. Dis. Model. Mech..

[231] Bloom G.S., Luca F.C., Vallee R.B. (1985). Microtubule-associated protein 1B: Identification of a major component of the neuronal cytoskeleton. Proc. Natl. Acad. Sci. USA.

[232] Noble M., Lewis S.A., Cowan N.J. (1989). The microtubule binding domain of microtubule-associated protein MAP1B contains a repeated sequence motif unrelated to that of MAP2 and tau. J. Cell Biol..

[233] Parysek L.M., Wolosewick J.J., Olmsted J.B. (1984). MAP 4: A microtubule-associated protein specific for a subset of tissue microtubules. J. Cell Biol..

[234] Job D., Rauch C.T., Fischer E.H., Margolis R.L. (1982). Recycling of cold-stable microtubules: Evidence that cold stability is due to substoichiometric polymer blocks. Biochemistry.

[235] Guillaud L., Bosc C., Fourest-Lieuvin A., Denarier E., Pirollet F., Lafanechere L., Job D. (1998). STOP proteins are responsible for the high degree of microtubule stabilization observed in neuronal cells. J. Cell Biol..

[236] Bulinski J.C., Bossler A. (1994). Purification and characterization of ensconsin, a novel microtubule stabilizing protein. J. Cell Sci..

[237] Fukata Y., Itoh T.J., Kimura T., Menager C., Nishimura T., Shiromizu T., Watanabe H., Inagaki N., Iwamatsu A., Hotani H. (2002). CRMP-2 binds to tubulin heterodimers to promote microtubule assembly. Nat. Cell Biol..

[238] Hooikaas P.J., Martin M., Muhlethaler T., Kuijntjes G.J., Peeters C.A.E., Katrukha E.A., Ferrari L., Stucchi R., Verhagen D.G.F., van Riel W.E. (2019). MAP7 family proteins regulate kinesin-1 recruitment and activation. J. Cell Biol..

[239] Lee G., Cowan N., Kirschner M. (1988). The primary structure and heterogeneity of tau protein from mouse brain. Science.

[240] Himmler A., Drechsel D., Kirschner M.W., Martin D.W. (1989). Tau consists of a set of proteins with repeated C-terminal microtubule-binding domains and variable N-terminal domains. Mol. Cell Biol..

[241] Kar S., Fan J., Smith M.J., Goedert M., Amos L.A. (2003). Repeat motifs of tau bind to the insides of microtubules in the absence of taxol. EMBO J..

[242] Lewis S.A., Wang D.H., Cowan N.J. (1988). Microtubule-associated protein MAP2 shares a microtubule binding motif with tau protein. Science.

[243] Baas P.W., Qiang L. (2019). Tau: It’s Not What You Think. Trends Cell Biol..

[244] Qiang L., Sun X., Austin T.O., Muralidharan H., Jean D.C., Liu M., Yu W., Baas P.W. (2018). Tau Does Not Stabilize Axonal Microtubules but Rather Enables Them to Have Long Labile Domains. Curr. Biol..

[245] Sun X., Yu W., Baas P.W., Toyooka K., Qiang L. (2024). Antagonistic roles of tau and MAP6 in regulating neuronal development. J. Cell Sci..

[246] Kosik K.S., Finch E.A. (1987). MAP2 and tau segregate into dendritic and axonal domains after the elaboration of morphologically distinct neurites: An immunocytochemical study of cultured rat cerebrum. J. Neurosci..

[247] Kanai Y., Hirokawa N. (1995). Sorting mechanisms of tau and MAP2 in neurons: Suppressed axonal transit of MAP2 and locally regulated microtubule binding. Neuron.

[248] Togel M., Wiche G., Propst F. (1998). Novel features of the light chain of microtubule-associated protein MAP1B: Microtubule stabilization, self interaction, actin filament binding, and regulation by the heavy chain. J. Cell Biol..

[249] Yang M., Wu M., Xia P., Wang C., Yan P., Gao Q., Liu J., Wang H., Duan X., Yang X. (2012). The role of microtubule-associated protein 1B in axonal growth and neuronal migration in the central nervous system. Neural Regen. Res..

[250] Noiges R., Eichinger R., Kutschera W., Fischer I., Nemeth Z., Wiche G., Propst F. (2002). Microtubule-associated protein 1A (MAP1A) and MAP1B: Light chains determine distinct functional properties. J. Neurosci..

[251] Mohan R., John A. (2015). Microtubule-associated proteins as direct crosslinkers of actin filaments and microtubules. IUBMB Life.

[252] Ding J., Valle A., Allen E., Wang W., Nardine T., Zhang Y., Peng L., Yang Y. (2006). Microtubule-associated protein 8 contains two microtubule binding sites. Biochem. Biophys. Res. Commun..

[253] Liu L., Vo A., Liu G., McKeehan W.L. (2005). Distinct structural domains within C19ORF5 support association with stabilized microtubules and mitochondrial aggregation and genome destruction. Cancer Res..

[254] West R.R., Tenbarge K.M., Olmsted J.B. (1991). A model for microtubule-associated protein 4 structure. Domains defined by comparisons of human, mouse, and bovine sequences. J. Biol. Chem..

[255] Olmsted J.B., Asnes C.F., Parysek L.M., Lyon H.D., Kidder G.M. (1986). Distribution of MAP-4 in cells and in adult and developing mouse tissues. Ann. N. Y. Acad. Sci..

[256] Bosc C., Oenarier E., Andrieux A., Job D. (1999). STOP proteins. Cell Struct. Funct..

[257] Adler A., Bangera M., Beugelink J.W., Bahri S., van Ingen H., Moores C.A., Baldus M. (2024). A structural and dynamic visualization of the interaction between MAP7 and microtubules. Nat. Commun..

[258] Kikuchi K., Nakamura A., Arata M., Shi D., Nakagawa M., Tanaka T., Uemura T., Fujimori T., Kikuchi A., Uezu A. (2018). Map7/7D1 and Dvl form a feedback loop that facilitates microtubule remodeling and Wnt5a signaling. EMBO Rep..

[259] Pan X., Cao Y., Stucchi R., Hooikaas P.J., Portegies S., Will L., Martin M., Akhmanova A., Harterink M., Hoogenraad C.C. (2019). MAP7D2 Localizes to the Proximal Axon and Locally Promotes Kinesin-1-Mediated Cargo Transport into the Axon. Cell Rep..

[260] Quach T.T., Honnorat J., Kolattukudy P.E., Khanna R., Duchemin A.M. (2015). CRMPs: Critical molecules for neurite morphogenesis and neuropsychiatric diseases. Mol. Psychiatry.

[261] Shao J., Zhang R., Wu P., Xu H., Zhou Z., Chen W., Gu L., Wang L., Luo S., Ren J. (2026). Systematic identification of microtubule lumen proteins reveals a taxane-sensitive luminal resident JPT2 regulating MEC17 accessibility. Proc. Natl. Acad. Sci. USA.

[262] Travis S.M., Kraus J., McManus C.T., Golden K., Zhang R., Petry S. (2025). How augmin establishes the angle of the microtubule branch site. Nat. Commun..

[263] Hutchins J.R., Toyoda Y., Hegemann B., Poser I., Heriche J.K., Sykora M.M., Augsburg M., Hudecz O., Buschhorn B.A., Bulkescher J. (2010). Systematic analysis of human protein complexes identifies chromosome segregation proteins. Science.

[264] Lawo S., Bashkurov M., Mullin M., Ferreria M.G., Kittler R., Habermann B., Tagliaferro A., Poser I., Hutchins J.R., Hegemann B. (2009). HAUS, the 8-subunit human Augmin complex, regulates centrosome and spindle integrity. Curr. Biol..

[265] Liu T., Tian J., Wang G., Yu Y., Wang C., Ma Y., Zhang X., Xia G., Liu B., Kong Z. (2014). Augmin triggers microtubule-dependent microtubule nucleation in interphase plant cells. Curr. Biol..

[266] Gabel C.A., Li Z., DeMarco A.G., Zhang Z., Yang J., Hall M.C., Barford D., Chang L. (2022). Molecular architecture of the augmin complex. Nat. Commun..

[267] Agostini L., Pfister J.A., Basnet N., Ding J., Zhang R., Biertumpfel C., O’Connell K.F., Mizuno N. (2025). Structural insights into SSNA1 self-assembly and its microtubule binding for centriole maintenance. Nat. Commun..

[268] Goyal U., Renvoise B., Chang J., Blackstone C. (2014). Spastin-interacting protein NA14/SSNA1 functions in cytokinesis and axon development. PLoS ONE.

[269] Basnet N., Nedozralova H., Crevenna A.H., Bodakuntla S., Schlichthaerle T., Taschner M., Cardone G., Janke C., Jungmann R., Magiera M.M. (2018). Direct induction of microtubule branching by microtubule nucleation factor SSNA1. Nat. Cell Biol..

[270] Tymanskyj S.R., Ma L. (2019). MAP7 Prevents Axonal Branch Retraction by Creating a Stable Microtubule Boundary to Rescue Polymerization. J. Neurosci..

[271] Tymanskyj S.R., Yang B., Falnikar A., Lepore A.C., Ma L. (2017). MAP7 Regulates Axon Collateral Branch Development in Dorsal Root Ganglion Neurons. J. Neurosci..

[272] Brouhard G.J., Rice L.M. (2018). Microtubule dynamics: An interplay of biochemistry and mechanics. Nat. Rev. Mol. Cell Biol..

[273] Baas P.W., Qiang L. (2005). Neuronal microtubules: When the MAP is the roadblock. Trends Cell Biol..

[274] Sferra A., Nicita F., Bertini E. (2020). Microtubule Dysfunction: A Common Feature of Neurodegenerative Diseases. Int. J. Mol. Sci..

[275] Barbiero I., De Rosa R., Kilstrup-Nielsen C. (2019). Microtubules: A Key to Understand and Correct Neuronal Defects in CDKL5 Deficiency Disorder?. Int. J. Mol. Sci..

[276] Gerdes J.M., Katsanis N. (2005). Microtubule transport defects in neurological and ciliary disease. Cell Mol. Life Sci..

[277] Prosser S.L., Pelletier L. (2017). Mitotic spindle assembly in animal cells: A fine balancing act. Nat. Rev. Mol. Cell Biol..

[278] Liu X., Pacwa A., Bresciani G., Swierczynska M., Dorecka M., Smedowski A. (2024). Retinal primary cilia and their dysfunction in retinal neurodegenerative diseases: Beyond ciliopathies. Mol. Med..

[279] Van De Weghe J.C., Gomez A., Doherty D. (2022). The Joubert-Meckel-Nephronophthisis Spectrum of Ciliopathies. Annu. Rev. Genom. Hum. Genet..

[280] Serpieri V., D’Abrusco F., Valente E.M. (2025). The relevance of primary cilia in neurological disorders. Lancet Neurol..

[281] Mill P., Christensen S.T., Pedersen L.B. (2023). Primary cilia as dynamic and diverse signalling hubs in development and disease. Nat. Rev. Genet..

[282] Grossman-Haham I., Coudray N., Yu Z., Wang F., Zhang N., Bhabha G., Vale R.D. (2021). Structure of the radial spoke head and insights into its role in mechanoregulation of ciliary beating. Nat. Struct. Mol. Biol..

[283] Moran A.L., Louzao-Martinez L., Norris D.P., Peters D.J.M., Blacque O.E. (2024). Transport and barrier mechanisms that regulate ciliary compartmentalization and ciliopathies. Nat. Rev. Nephrol..

[284] Klena N., Pigino G. (2022). Structural Biology of Cilia and Intraflagellar Transport. Annu. Rev. Cell Dev. Biol..

[285] Lingle W.L., Salisbury J.L. (1999). Altered centrosome structure is associated with abnormal mitoses in human breast tumors. Am. J. Pathol..

[286] Nigg E.A. (2006). Origins and consequences of centrosome aberrations in human cancers. Int. J. Cancer.

[287] Godinho S.A., Pellman D. (2014). Causes and consequences of centrosome abnormalities in cancer. Philos. Trans. R. Soc. Lond. B Biol. Sci..

[288] Raff J.W., Basto R. (2017). Centrosome Amplification and Cancer: A Question of Sufficiency. Dev. Cell.

[289] Kiermaier E., Stotzel I., Schapfl M.A., Villunger A. (2024). Amplified centrosomes-more than just a threat. EMBO Rep..

[290] Ganem N.J., Godinho S.A., Pellman D. (2009). A mechanism linking extra centrosomes to chromosomal instability. Nature.

[291] Levine M.S., Bakker B., Boeckx B., Moyett J., Lu J., Vitre B., Spierings D.C., Lansdorp P.M., Cleveland D.W., Lambrechts D. (2017). Centrosome Amplification Is Sufficient to Promote Spontaneous Tumorigenesis in Mammals. Dev. Cell.

[292] Sercin O., Larsimont J.C., Karambelas A.E., Marthiens V., Moers V., Boeckx B., Le Mercier M., Lambrechts D., Basto R., Blanpain C. (2016). Transient PLK4 overexpression accelerates tumorigenesis in p53-deficient epidermis. Nat. Cell Biol..

[293] Fong C.S., Mazo G., Das T., Goodman J., Kim M., O’Rourke B.P., Izquierdo D., Tsou M.F. (2016). 53BP1 and USP28 mediate p53-dependent cell cycle arrest in response to centrosome loss and prolonged mitosis. eLife.

[294] Basto R., Lau J., Vinogradova T., Gardiol A., Woods C.G., Khodjakov A., Raff J.W. (2006). Flies without centrioles. Cell.

[295] Barbier P., Zejneli O., Martinho M., Lasorsa A., Belle V., Smet-Nocca C., Tsvetkov P.O., Devred F., Landrieu I. (2019). Role of Tau as a Microtubule-Associated Protein: Structural and Functional Aspects. Front. Aging Neurosci..

[296] de Forges H., Bouissou A., Perez F. (2012). Interplay between microtubule dynamics and intracellular organization. Int. J. Biochem. Cell Biol..

[297] Wattanathamsan O., Pongrakhananon V. (2022). Emerging role of microtubule-associated proteins on cancer metastasis. Front. Pharmacol..

[298] Bhat K.M., Setaluri V. (2007). Microtubule-associated proteins as targets in cancer chemotherapy. Clin. Cancer Res..

[299] Perez de Castro I., Malumbres M. (2012). Mitotic Stress and Chromosomal Instability in Cancer: The Case for TPX2. Genes Cancer.

[300] Polverino F., Mastrangelo A., Guarguaglini G. (2024). Contribution of AurkA/TPX2 Overexpression to Chromosomal Imbalances and Cancer. Cells.

[301] Brüning-Richardson A., Bond J., Alsiary R., Richardson J., Cairns D.A., McCormac L. (2012). NuMA Overexpression in Epithelial Ovarian Cancer. PLoS ONE.

[302] Wang Q., Chen Y., Feng H., Zhang B., Wang H. (2018). Prognostic and predictive value of HURP in non-small cell lung cancer. Oncol. Rep..

[303] Kreis N.-N., Moon H.H., Wordeman L., Louwen F., Solbach C., Yuan J., Ritter A. (2024). KIF2C/MCAK a prognostic biomarker and its oncogenic potential in malignant progression, and prognosis of cancer patients: A systematic review and meta-analysis as biomarker. Crit. Rev. Clin. Lab. Sci..

[304] Li J., Hubisz M.J., Earlie E.M., Duran M.A., Hong C., Varela A.A., Lettera E., Deyell M., Tavora B., Havel J.J. (2023). Non-cell-autonomous cancer progression from chromosomal instability. Nature.

[305] Zhang D., Dai L., Yang Z., Wang X., LanNing Y. (2019). Association of STMN1 with survival in solid tumors: A systematic review and meta-analysis. Int. J. Biol. Markers.

[306] Sugihara Y., Taniguchi H., Kushima R., Tsuda H., Kubota D., Ichikawa H., Sakamoto K., Nakamura Y., Tomonaga T., Fujita S. (2012). Proteomic-based identification of the APC-binding protein EB1 as a candidate of novel tissue biomarker and therapeutic target for colorectal cancer. J. Proteom..

[307] Berges R., Baeza-Kallee N., Tabouret E., Chinot O., Petit M., Kruczynski A., Figarella-Branger D., Honore S., Braguer D. (2014). End-binding 1 protein overexpression correlates with glioblastoma progression and sensitizes to Vinca-alkaloids in vitro and in vivo. Oncotarget.

[308] Zaoui K., Duhamel S., Parachoniak C.A. (2019). CLIP-170 spatially modulates receptor tyrosine kinase recycling to coordinate cell migration. Traffic.

[309] Saju A., Chen P.-P., Weng T.-H., Tsai S.-Y., Tanaka A., Tseng Y.-T., Chang C.-C., Wang C.-H., Shimamoto Y. (2024). HURP binding to the vinca domain of β-tubulin accounts for cancer drug resistance. Nat. Commun..

[310] Thakkar P.V., Kita K., Castillo U.D., Galletti G., Madhukar N., Navarro E.V., Barasoain I., Goodson H.V., Sackett D., Diaz J.F. (2021). CLIP-170S is a microtubule +TIP variant that confers resistance to taxanes by impairing drug-target engagement. Dev. Cell.

[311] Bai T., Yokobori T., Altan B., Ide M., Mochiki E., Yanai M., Kimura A., Kogure N., Yanoma T., Suzuki M. (2017). High STMN1 level is associated with chemo-resistance and poor prognosis in gastric cancer patients. Br. J. Cancer.

[312] Cushion T.D., Leca I., Keays D.A. (2023). MAPping tubulin mutations. Front. Cell Dev. Biol..

[313] Spillantini M.G., Goedert M. (2013). Tau pathology and neurodegeneration. Lancet Neurol..

[314] Strang K.H., Golde T.E., Giasson B.I. (2019). MAPT mutations, tauopathy, and mechanisms of neurodegeneration. Lab. Investig..

[315] Cario A., Berger C.L. (2023). Tau, microtubule dynamics, and axonal transport: New paradigms for neurodegenerative disease. Bioessays.

[316] Creekmore B.C., Watanabe R., Lee E.B. (2024). Neurodegenerative Disease Tauopathies. Annu. Rev. Pathol..

[317] Bakota L., Tulva K., Trushina N.I., Brandt R. (2026). Beyond microtubule regulation: The multifaceted roles of tau in neuronal function and dysfunction. Transl. Psychiatry.

[318] Gleeson J.G., Allen K.M., Fox J.W., Lamperti E.D., Berkovic S., Scheffer I., Cooper E.C., Dobyns W.B., Minnerath S.R., Ross M.E. (1998). Doublecortin, a brain-specific gene mutated in human X-linked lissencephaly and double cortex syndrome, encodes a putative signaling protein. Cell.

[319] des Portes V., Francis F., Pinard J.M., Desguerre I., Moutard M.L., Snoeck I., Meiners L.C., Capron F., Cusmai R., Ricci S. (1998). doublecortin is the major gene causing X-linked subcortical laminar heterotopia (SCLH). Hum. Mol. Genet..

[320] Cuveillier C., Boulan B., Ravanello C., Denarier E., Deloulme J.C., Gory-Faure S., Delphin C., Bosc C., Arnal I., Andrieux A. (2021). Beyond Neuronal Microtubule Stabilization: MAP6 and CRMPS, Two Converging Stories. Front. Mol. Neurosci..

